# Self-Assembled Carrier-Free Nanostructures of Anemoside B4 and Oleanolic Acid Alleviate Intestinal Injury Induced by Intraperitoneal *Escherichia coli* Challenge in Mice

**DOI:** 10.3390/antiox15091081

**Published:** 2026-08-28

**Authors:** Jianchi Lun, Yuxin Yan, Junji Huang, Rong Chen, Yimu Ma, Mengjie Liu, Qian Qu, Weijie Lv, Shining Guo

**Affiliations:** 1 College of Veterinary Medicine, South China Agricultural University, Guangzhou 510642, China; ljc13112702335@163.com (J.L.);; 2 School of Agriculture and Biology, Shanghai Jiao Tong University, Shanghai 200240, China; 3 Shanghai Laboratory Animal Research Center, Shanghai 201203, China; 4 School of Animal Science and Technology, Foshan University, Foshan 528000, China; 5 Guangdong Technology Research Center for Traditional Chinese Veterinary Medicine and Natural Medicine, Guangzhou 510642, China

**Keywords:** anemoside B4, oleanolic acid, *Escherichia coli*, nanoparticles, gut microbiota

## Abstract

*Escherichia coli*-induced enteritis imposes a substantial economic burden on the global livestock industry. In the current context of reducing and restricting antibiotic use, there is an urgent need for novel therapeutic strategies. Based on traditional Chinese medicine compatibility, anemoside B4 (AB4) and oleanolic acid (OA) form nanoscale assemblies (AB4-OA NPs) through hydrogen bonds and van der Waals forces. Physicochemical characterization quantified the NPs as spherical particles with an average hydrodynamic diameter of 164.16 nm, a PDI of 0.280, zeta potential of −22.16 mV, and stable particle size for 28 days; the hemolysis ratio remained below 3.38% at concentrations up to 2 mg/mL, confirming favorable biosafety. In vitro assays confirmed that AB4-OA NPs outperformed AB4/OA physical mixtures in anti-inflammatory and antioxidant activity by restraining pro-inflammatory factors and activating antioxidant enzymes, verifying nanotechnology’s potency in boosting their bioactivity. In mice challenged intraperitoneally with *E. coli*, AB4-OA NPs elevated SOD, CAT, GSH-Px and lowered MDA; they inhibited the NF-κB pathway, activated the Nrf2 pathway, balanced inflammatory cytokines, alleviated intestinal leakage, upregulated tight junction proteins and reshaped gut microbiota by reducing *Proteobacteria* and enriching *Firmicutes*. Spearman’s correlation study indicated a strong positive relationship between *Lactobacillus* and *Faecalibacterium* with intestinal barrier function (Occludin and ZO-1) as well as antioxidant capacity, while *Enterococcus* was significantly and positively correlated with pro-inflammatory cytokines. These findings demonstrate that AB4-OA NPs exert pronounced protective therapeutic effects on *E. coli*-induced enteritis via inhibition of inflammation, attenuation of oxidative stress, protection of the intestinal barrier, and regulation of gut microbiota, and that these effects were greater than those of the corresponding physical mixture. This study proposes a novel therapeutic strategy for managing *E. coli*-induced enteritis and highlights the therapeutic potential of phytochemical-based self-assembled nanomedicines.

## 1. Introduction

The global livestock and poultry industry faces substantial economic losses attributable to bacterial infections, with enteritis caused by *Escherichia coli* (*E. coli*) being one of the most significant contributors [1]. Beyond the direct costs of treatment, these infections severely compromise critical production parameters, including growth rate and feed conversion efficiency [2]. For instance, *E. coli* infection has been reported to reduce average daily feed intake (ADFI) by 10%, culminating in a 28% decrease in average daily gain (ADG), thereby imposing a considerable burden on poultry production [3]. Watery diarrhea and intestinal secretory function can be directly impacted by an *E. coli* infection. Excessive inflammatory reactions and an imbalance in oxidative stress are directly linked to its fundamental pathogenic process [4,5]. *E. coli* and its lipopolysaccharide (LPS) can activate host innate immunity, induce massive release of pro-inflammatory cytokines, and trigger a cascade of inflammatory responses [6,7]. Meanwhile, they cause excessive accumulation of reactive oxygen species, disrupt the oxidant–antioxidant balance, aggravate intestinal epithelial cell injury and mucosal barrier leakage, and ultimately lead to typical symptoms such as diarrhea, fever, intestinal mucosal damage, and systemic inflammation [8]. At present, antibiotic therapy remains the mainstay for controlling avian colibacillosis in commercial poultry; however, the escalating prevalence of multidrug-resistant *E. coli* strains, together with concerns over drug residues and antibiotic-induced disruption of the intestinal microbiota, has driven an urgent search for safe and effective antibiotic-sparing alternatives, among which phytochemical-based formulations have attracted particular interest [9]. Elucidating how natural products counteract *E. coli*-induced intestinal injury is therefore of direct relevance to sustainable poultry production. Accordingly, in established mouse models of enterotoxigenic *E. coli* (ETEC) infection, measurable decreases in body weight, increased diarrhea incidence, and elevated intestinal inflammatory markers are typically observed within 3 days of challenge [10,11]. Therefore, a 3-day post-infection observation window was selected in the present study to capture these acute responses.

Decoctum Pulsatillae was initially described by Zhang Zhongjing in the Treatise on Cold Damage (Shang Han Lun) [12]. This is a traditional Chinese medicine formula commonly used in clinical settings to treat diarrhea caused by *E. coli* infection [13]. However, the formulation’s complexity, difficulties in standardizing ingredients, compositional profile, and a lack of knowledge about its therapeutic material foundation restrict its wide clinical applicability [14]. Among its constituents, Anemoside B4 (AB4) and Oleanolic Acid (OA), two bioactive phytochemicals isolated from Decoctum Pulsatillae, have been separately validated to exert anti-inflammatory, antioxidant and mild antibacterial effects in previous pharmacological investigations [15,16]. Nevertheless, two intrinsic physicochemical bottlenecks severely restrict their in vivo therapeutic performance: AB4 possesses a large glycosidic structure that results in poor intestinal membrane permeability and low absorption efficiency; OA exhibits strong lipophilicity and extremely low water solubility, leading to rapid metabolic clearance and limited local retention in intestinal lesions. Recent studies have further linked these activities to intestinal protection: AB4 attenuates experimental colitis and lipopolysaccharide-induced intestinal epithelial injury by suppressing NF-κB-mediated inflammation and oxidative stress [17], whereas OA alleviates intestinal inflammation and preserves epithelial barrier integrity in models of intestinal injury [18]. Notably, AB4 is a triterpenoid saponin whose hydrophilic sugar chains are attached to a hydrophobic pentacyclic triterpene skeleton, whereas OA is a lipophilic pentacyclic triterpene acid; the two compounds therefore provide complementary hydrophilic and hydrophobic properties that have not yet been exploited therapeutically.

Recent advances in the interdisciplinary study of nanoscience and Traditional Chinese Medicine (TCM) have revealed that herbal decoctions contain not only water-soluble chemicals but also various nano-sized particles [19]. According to studies, the presence of supramolecular aggregates in these decoctions might greatly improve the pharmacodynamic efficacy of the active substances by increasing their stability, solubility, and gastrointestinal absorption [20,21,22]. This principle is exemplified by self-assembled nanoparticles derived from TCM formulations, which have been demonstrated to effectively improve oral bioavailability. For instance, in the self-assembled systems associated with Shaoyao Gancao Decoction, glycyrrhizic acid self-assembled nanomicelles (GL-SNM) and glycyrrhizin protein self-assembled nanoparticles (GP-SAN) increased the bioavailability of paeoniflorin by 65% and 45%, respectively [23]. Building upon this foundation, herbal pair-derived self-assembled nanostructures—grounded in TCM compatibility theory—have emerged as a particularly attractive research direction. Unlike conventional single-component nanoformulations, these systems enable the co-delivery of two synergistic bioactive compounds without the need for additional excipients [24]. A growing body of research has further advanced this concept through amphiphile-driven core–shell supramolecular self-assembly, which represents a frontier in oral nanomedicine delivery. Recent state-of-the-art studies employing molecular dynamics simulations and multi-spectral characterization (FT-IR, ^1^H NMR) have elucidated that glycoside triterpenoid composites adopt ordered core–shell architectures: hydrophilic sugar side chains extend into the aqueous phase to form an anionic outer shell, while lipophilic aglycone fragments aggregate inward to construct a hydrophobic core capable of encapsulating poorly soluble partner drugs [25,26]. This unique structural organization confers distinct advantages to the formulation, including sustained drug release and electrostatic targeting capability. Beyond these formulation-derived examples, amphiphilic triterpenoid saponins such as ginsenosides have also been widely exploited as natural building blocks of self-assembled nanocarriers, usually for the delivery of a single therapeutic agent [27], confirming that saponins can drive stable nanoaggregate formation through hydrophobic interactions among aglycone moieties and hydrogen bonding among sugar chains. These insights provide fresh viewpoints for understanding the material foundation and mechanisms of TCM formulations. These insights provide fresh viewpoints for understanding the material foundation and mechanisms of TCM formulations. However, current research on herbal self-assembling nanoparticles has predominantly focused on flavonoids, alkaloids, or polysaccharide-based carriers [28,29]. Few studies have employed amphiphilic triterpenoid saponins as natural assembly scaffolds for the co-loading of hydrophobic triterpene acids, and the intermolecular driving forces and conformational changes underlying the formation of saponin-triterpene binary nanoaggregates remain poorly understood. More importantly, no carrier-free self-assembled system based on a triterpenoid saponin and its hydrophobic triterpene acid aglycone has been developed or evaluated for the oral treatment of bacterial enteritis in poultry.

In this study, we focus on the small molecular components of Pulsatilla decoction. Structurally, AB4 contains two hydrophilic sugar side chains and a hydrophobic pentacyclic triterpene aglycone, enabling it to self-assemble into nanostructures in aqueous environments. This property allows AB4 to improve the solubility of hydrophobic drugs and suggests promising potential as a novel nanomedicine delivery platform. In contrast, OA suffers from poor water solubility that limits its in vivo effectiveness. Leveraging self-assembly principles, we constructed a co-delivery nanoparticle system through the spontaneous assembly of AB4 and OA. The present approach aims to provide a green, low-toxicity carrier-free nanotherapeutic candidate for livestock bacterial enteritis, and unveils the supramolecular material basis of classic TCM herbal pairs, offering novel mechanistic evidence for modern interpretation of TCM compatibility rules.

## 2. Materials and Methods

### 2.1. Preparation and Characterization of AB4-OA NPs

AB4-OA was synthesized using an anti-solvent approach. To put it briefly, 10 mg of OA was dissolved in 10 mL of anhydrous ethanol and 10 mg of AB4 was dissolved in 10 mL of ultrapure water. Then, 1 mL of the OA ethanol solution was added dropwise to 9 mL of the AB4 aqueous solution, and the mixture was stirred for 15 min. The mixture was then ultrasonicated for 30 min. A dialysis bag was then used to dialyze the crude product against ultrapure H_2_O for 24 h in order to extract the organic solvent and release the free medication. A transmission electron microscope (TEM, FEI, Brno, Czech Republic) and a scanning electron microscope (SEM, FEI, Brno, Czech Republic) were used to examine the size and shape of AB4-OA nanoparticles. A Zetasizer Nano ZS (Malvern Instruments, Worcestershire, UK) was used to evaluate the hydrodynamic diameters and zeta potential of AB4-OA nanoparticles. The chemical structures of AB4-OA NPs were analyzed using a Fourier transform infrared (FT-IR) spectrometer (Thermo Fisher, Nicolet IS10, Madison, WI, USA) and nuclear magnetic resonance (NMR) (Bruker, AVANCE NEO 600, Rheinstetten, Germany). Three matched control samples were prepared in parallel with consistent drug concentrations and the AB4:OA mass ratio of 10:1 for comparative analysis: free AB4 aqueous solution containing an equal amount of AB4 without OA, free OA solvent solution with the equivalent OA concentration, and AB4/OA physical mixture prepared by directly mixing AB4 and OA powders at the same proportion without nanoprecipitation or ultrasonic self-assembly treatment. The contents of AB4 and OA in AB4-OA NPs were determined using an HPLC system (Shimadzu, Kyoto, Japan) consisting of a tunable absorbance detector. To determine AB4, the mobile phase was made up of acetonitrile and water in a 29 to 71 volume ratio. The wavelength used for detection was 201 nm, utilizing a Waters SunFire C18 column with a flow rate of 1 mL/min and a column temperature set at 35 °C. To determine OA, the mobile phase was made up of acetonitrile and water in a 90:10 volume ratio. A Waters SunFire C18 column detected at a wavelength of 205 nm while running at a flow rate of 1 mL/min. The column temperature was maintained at 25 °C. To determine encapsulation efficiency (EE%) and drug loading capacity (DLC%), the freeze-dried products were dissolved in methanol, then the supernatant was collected by centrifugation at room temperature (10,000 rpm for 5 min) for measuring its AB4 and OA concentrations.

(1) $$\mathrm{EE}\;(\%)=\frac{\mathrm{Weight}\;\mathrm{of}\;\mathrm{loaded}\;\mathrm{drug}}{\mathrm{Theoretical}\;\mathrm{drug}\;\mathrm{loaded}}\;\times \;100\%$$

(2) $$\mathrm{DLC}\;(\%)=\frac{\mathrm{Weight}\;\mathrm{of}\;\mathrm{loaded}\;\mathrm{drug}}{\mathrm{Weight}\;\mathrm{of}\;\mathrm{nanomicelles}}\;\times \;100\%$$

### 2.2. Molecular Dynamics Simulation of AB4-OA NPs

Using Packmol software (20.15.3, University of Campinas, Campinas, Brazil), two molecular mixtures were created at a 2:1 molar ratio. The resulting mixture was then solvated in an 8 × 8 × 8 nm simulation box filled with TIP3P water molecules. Long-range electrostatic interactions were handled using the Particle Mesh Ewald (PME) method. The OPLS-AA force field was used to define the complete system. The solvent was first energy-minimized and then equilibrated in the NVT ensemble for 1 ps using the V-rescale thermostat at 298.15 K, followed by an NPT equilibration for 2 ps with the Parrinello-Rahman barostat at 1 bar. Finally, all bonds containing hydrogen atoms were constrained using the LINCS algorithm during a 200 ns MD production simulation with a 2 fs time step. The GROMACS software package (2024.5, University of Groningen, Groningen, The Netherlands) from the University of Groningen in the Netherlands was used for the MD simulations, while VMD 1.9.3 (University of Illinois at Urbana–Champaign, Urbana, IL, USA) from the University of Illinois Urbana–Champaign in the USA was employed for visualizations [30].

### 2.3. In Vitro Drug Release of AB4-OA NPs

The dialysis technique in PBS (pH 7.4) with 0.1% (*v*/*v*) Tween 80 was used to examine the release characteristics of AB4 and OA from AB4-OA NPs. In short, 500 mL of buffer was placed outside dialysis bags (MWCO 3500) containing 10 mL of AB4-OA NPs, which were then incubated at 37 °C while being agitated at 100 rpm. 100 μL of dialysis fluid was taken out of the dialysis bag at predetermined intervals. HPLC was used to measure the amount of released AB4 and OA in the supernatant, respectively [31].

### 2.4. Hemolysis Assay of AB4-OA NPs

Mouse erythrocytes were used in a hemolysis test of AB4-OA NPs. Fresh whole blood was collected from healthy mice via retro-orbital venous plexus puncture. The blood sample was centrifuged to separate the plasma and leukocyte layer. After carefully aspirating and discarding the supernatant (plasma and the uppermost leukocyte buffy coat), the remaining erythrocyte pellet was resuspended in pre-cooled 0.9% saline and subjected to three repeated centrifugation–washing cycles. The purified erythrocytes were then diluted with 0.9% saline to prepare a 2% (*v*/*v*) RBC suspension for subsequent experiments. For the hemolysis assay, serially diluted nanoparticle stock solutions were mixed with equal volumes of the RBC suspension, yielding final nanoparticle concentrations ranging from 0.5 to 2 mg/mL in the reaction mixture. Ultrapure water acted as the positive control, and normal saline acted as the negative control. After two hours of incubation at 37 °C, the RBC suspension was measured at 540 nm using a microplate reader. A nanoparticle control group was set up, in which an equal concentration gradient of AB4-OA NPs was incubated in 0.9% saline without erythrocytes under identical conditions. To eliminate the optical interference caused by the nanoparticles themselves, the absorbance of a blank control containing the corresponding concentration of nanoparticles was subtracted from the absorbance of each experimental sample. The hemolysis ratio of AB4-OA NPs was calculated by Equation (3):

(3) $$\mathrm{Hemolysis}\;\mathrm{rate}\;(\%)=\frac{\mathrm{OD}\;(\mathrm{Sample})\;-\;\mathrm{OD}\;(\mathrm{Negative}\;\mathrm{control})}{\mathrm{OD}\;(\mathrm{Positive}\;\mathrm{control})\;-\;\mathrm{OD}\;(\mathrm{Negative}\;\mathrm{control})}\;\times \;100\%$$

### 2.5. In Vitro Anti-Inflammatory Effect

The RAW264.7 macrophage cell line originates from the China Center for the Preservation of Typical Cultures. RAW264.7 cells were cultured using Dulbecco’s Modified Eagle Medium (DMEM) (Gibco) and Fetal Bovine Serum (FBS) (Thermo Fisher Scientific, Waltham, MA, USA). Cultivate in a constant humidity environment at 37 °C and 5% carbon dioxide. RAW264.7 cells were grown in DMEM with 10% fetal bovine serum (37 °C, 5% CO_2_) and divided into 5 groups (*n* = 3): CON group: Normal culture, no treatment; LCON group: Treated with 1 μg/mL lipopolysaccharide (LPS, Sigma-Aldrich, St. Louis, MO, USA) for 6 h; LMNPS group: After modeling with LPS, treatment with 625 μg/mL AB4-OA NPs for 12 h; LHNPS group: After modeling with LPS, treatment with 1250 μg/mL AB4-OA NPs for 12 h; LAB4@OA group: After LPS stimulation, a physical mixture of 1250 μg/mL AB4 and OA (mass ratio 10:1) was treated for 12 h. The Cell Counting Kit-8 method (Biosharp, Hefei, China) was employed to assess cell viability, while the Griess method (Beyotime Biotechnology, Shanghai, China) was used to measure Nitric Oxide (NO) levels in the supernatant. The levels of Interleukin-1 beta (IL-1β), Interleukin-6 (IL-6), TNF-α (Tumor Necrosis Factor-alpha), and IL-10 (Interleukin-10) were measured using ELISA (Shanghai Enzyme-linked Biotechnology Co., Ltd., Shanghai, China). The mRNA expressions of inducible nitric oxide synthase (iNOS), toll-like receptor 4 (TLR4), myeloid differentiation primary response protein 88 (MyD88), and P65 were detected by qRT-PCR (Analytik Jena, Jena, Germany) [32].

### 2.6. In Vitro Antioxidant Effect

RAW264.7 cells were seeded and grouped following the procedures described in Section 2.5. After 12 h of incubation with AB4-OA NPs or the physical mixture, Cell lysis buffer was added on ice after the cells had been washed once with PBS that had been chilled beforehand. Using a cell scraper, the cells were carefully removed and put into 1.5 mL EP tubes. The intracellular supernatants were obtained following centrifugation at 10,000 rpm for 5 min at 4 °C. The levels of malondialdehyde (MDA), as well as the activities of superoxide dismutase (SOD) and catalase (CAT), were determined using commercial assay kits (Jiancheng Bioengineering Institute, Nanjing, China) according to the manufacturer’s instructions. The protein concentration was determined using a BCA protein assay kit.

### 2.7. AB4-OA NPs for Bacterial Enteritis Treatment

All procedures involving animals received approval from the Animal Ethics Committee at South China Agricultural University (registration number: 2024f368, Guangzhou, China). The mice were accommodated and looked after at the South China Agricultural University’s animal experiment center (permission number: SYXK 2024–0136). Male SPF-grade C57BL/6 mice, purchased from Guangdong Zhiyuan Biomedical Technology Co., Ltd., weighing 17–18 g, were adaptively raised for one week and then randomly divided into 6 groups, with 12 mice in each group and 6 mice in each cage. Six groups of mice were used: CON (intact control, vehicle treatment), ECON (ETEC-challenged control, vehicle treatment), ELNPS, EMNPS, and EHNPS (ETEC-challenged mice treated with low (12.5 mg/kg), medium (25 mg/kg), and high-dose (50 mg/kg) AB4-OA NPs, respectively), and EAB4@OA (ETEC-challenged mice treated with the physical mixture of free AB4 and OA at the equivalent high dose, 50 mg/kg).

The sample size was determined a priori from day-3 body-weight data of a pilot experiment conducted under the same husbandry and challenge conditions as the main study (pooled within-group SD ≈ 0.98 g; expected group means 16.40–17.83 g) [33]. To allow for the uncertainty of the small-sample estimate, a conservative common within-group SD of σ = 1.0 g was adopted. Using the adopted σ = 1.0 g and the expected group means, the effect size was calculated as Cohen’s f = 0.46 using Equation (4) (a large effect). With a two-sided significance level of α = 0.05 and a statistical power of 80%, the required total sample size was determined from the F-test power function implemented in G*Power 3.1 (Heinrich Heine University Düsseldorf, Düsseldorf, Germany), yielding a total of 72 mice (12 per group).

(4) $$\mathrm{Cohen}’s\;f=\sqrt{\frac{\Sigma {(\mu i-\bar{\mu })}^{2}}{\left(k\cdot \sigma^{2}\right)}}$$

Following adaptation, both the ECON and treatment groups received an intraperitoneal injection of 0.2 mL of sterile PBS with 1 × 10^8^ CFU/mL ETEC K88, whereas the blank group (CON) was administered the same volume of sterile PBS [10]. The general status of all ETEC-challenged mice was continuously observed throughout the post-infection period. Mice were treated with the corresponding therapeutic agents at 6 h post-ETEC challenge, by which time typical enteritis symptoms (including reduced feed intake, lethargy, and loose feces) had been confirmed in preliminary experiments. AB4-OA NPs (50, 25, and 12.5 mg/kg) and the physical mixture (50 mg/kg) were each suspended in ultrapure water and administered orally once daily for three consecutive days. The doses of AB4-OA NPs were selected based on two criteria: the 50 mg/kg dose was within the range of previously reported effective oral doses of AB4 and OA in mouse models of intestinal injury [18,34], and preliminary safety experiments confirmed that 50 mg/kg was well tolerated, with no mortality or observable adverse effects during the treatment period. The 25 and 12.5 mg/kg doses were included to assess the dose–response relationship, and the physical mixture was administered at the equivalent dose (50 mg/kg) to enable direct comparison with the nanoparticle formulation. Body weight, diarrhea incidence, and survival were monitored daily throughout the study. On day 3 post-challenge, all mice were euthanized, and blood, liver, spleen, jejunum, ileum, and cecum contents were collected for the analyses described in the corresponding subsections. Mice were housed under controlled conditions (temperature of 20–26 °C, humidity of 40–70%, 12-h light/dark cycle) with free access to food and water. To minimize pain, distress, and suffering, mice were monitored at least twice daily (morning and evening) throughout the experiment, and the following clinical signs were evaluated: body weight, food and water intake, general activity, posture, coat condition, diarrhea incidence and severity, signs of dehydration, and mortality. Predefined humane endpoints were established before the experiment, including: (1) body weight loss ≥ 20% relative to the initial body weight; (2) severe diarrhea accompanied by dehydration or lethargy; (3) inability to reach food or water; and (4) severe systemic illness. Any mouse reaching a humane endpoint was immediately euthanized by cervical dislocation under deep isoflurane anesthesia and recorded as having met the early euthanasia criterion. Supportive care, consisting of ad libitum food and water and maintenance of an appropriate ambient temperature, was provided throughout the study; no additional supportive treatment was administered. All procedures, including intraperitoneal challenge, oral gavage, blood collection, and euthanasia, were performed with minimal handling and restraint, and blood collection and euthanasia were performed under deep isoflurane anesthesia, consistent with the AVMA Guidelines for the Euthanasia of Animals (2020).

### 2.8. Clinical Symptoms and Sample Collection

Following *E. coli* challenge, body weight, diarrhea incidence, and survival rate were assessed daily. All mice were euthanized, and blood samples were collected on day 3 post-infection. Briefly, each mouse was first anesthetized via intraperitoneal injection of pentobarbital sodium (50 mg/kg body weight). Deep anesthesia was confirmed by the complete loss of corneal and toe-pinch reflexes. Blood samples (approximately 0.3–0.5 mL per mouse) were then collected via retro-orbital venous plexus puncture before the animals were euthanized by cervical dislocation [35]. The sampling method and volume were in accordance with previously described methods and published guidelines for blood removal from laboratory mice [36]. After euthanasia, fresh livers, spleens, and stool were collected to count the microorganisms. Two batches of tissue samples were collected from the jejunum and ileum, respectively: one portion was fixed in 4% paraformaldehyde (Beijing Boruichangyuan Technology Co., Ltd., Beijing, China), and the other was immediately snap-frozen and stored at −80 °C. Blood was centrifuged at 3500× *g* for 15 min at 4 °C using a Biofuge 22R centrifuge from Heraeus, Hanau, Germany, and the serum was extracted and stored at −80 °C for later analysis. The cecum contents were promptly placed on ice and kept at −80 °C for 16S rRNA analysis.

### 2.9. Liver, Spleen, and Fecal Microbiota Count

The total *E. coli* loads in the liver, spleen, and feces of mice were determined using the dilution plating technique. Sterile PBS was used to suspend liver, spleen, and fecal samples, and the cultures were vortexed (each fresh fecal sample of 0.5 g was combined with 4.5 mL of sterile saline). Subsequently, 100 µL homogenates were suitably diluted and spread on MacConkey agar plates to count the colonies. After incubating the plates for 24 h at 37 °C in an aerobic setting, the data were presented as CFU per gram of feces. The experiment was conducted three times [37].

### 2.10. Evaluation of Inflammatory, Oxidative, and Intestinal Barrier Markers

The concentrations of proinflammatory cytokines including Interleukin-6 (IL-6), Tumor necrosis factor-α (TNF-α) and Interleukin-10 (IL-10) were determined using corresponding ELISA kits (Nanjing Jiancheng Bioengineering Institute, Nanjing, China). The levels of Malondialdehyde (MDA), Catalase (CAT), Superoxide dismutase (SOD), D-lactic acid (DLA) and Diamine oxidase (DAO) were measured by spectrophotometric assay kits from the same manufacturer following the manufacturer’s instructions.

### 2.11. Hematoxylin and Eosin (H&E) Staining

Tissue samples (2 cm) were collected from the mid-ileum and mid-jejunum and fixed in 4% paraformaldehyde. The tissue was sliced, then dried, embedded in paraffin, and stained with eosin and hematoxylin. Media Cybernetics, MD, USA’s Image-Pro Plus 6.0 software was used to measure the villus height and crypt depth. Finally, the ratio of villus height to crypt depth (VH/CD) was calculated.

### 2.12. Tissue RNA Extraction and Analysis of Relative Expression Levels of Genes

Primers were specifically designed using Primer 6.0 software (PREMIER Biosoft International, Palo Alto, CA, USA) and synthesized by Shenggong Biotech Co., Ltd. in Shanghai, China (refer to Table S1). RNA was isolated from the tissues of the jejunum and ileum using a TRIzol kit from Invitrogen, Carlsbad, CA, USA. The concentration of RNA was measured with a NanoDrop Lite spectrophotometer from Thermo Fisher Scientific, located in Waltham, MA, USA. The HiScript II All-in-One RT SuperMix from Vazyme Biotech Co., Ltd., Nanjing, China, was used for cDNA synthesis. Quantitative real-time PCR (qRT-PCR) LightCycler^®^ 480 II Real-Time PCR System (Roche Diagnostics, Mannheim, Germany) with the ChamQ Universal SYBR qPCR Master Mix from Vazyme Biotech Co., Ltd. in Nanjing, China, in accordance with the manufacturer’s instructions. Target genes analyzed in this assay included TLR4, TNF-α, IL-6, IL-10, MyD88, iNOS, P65, CAT, GSH-Px, SOD1, HO-1, Keap-1, Nrf2, Muc2, ZO-1, Occludin, Claudin-1. β-actin was used as the endogenous reference gene. Relative mRNA expression levels were calculated using the 2^−ΔΔCt^ method [38].

### 2.13. 16S rRNA Sequencing and Gut Microbiota Analysis

Samples of fresh cecal content were gathered from *E. coli*-challenged mice in the three treatment groups (CON, ECON, and ENPS (ETEC-stimulated mice treated with high-dose (50 mg/mL) AB4-OA self-assembled NPs)) for analysis of cecal microbiota (*n* = 6), as previously mentioned [39]. Fresh cecal contents were selected because the mouse cecum is the major site of microbial fermentation with the highest density and diversity of the gut microbiota, and because, unlike fecal and other intestinal contents that are markedly diluted during ETEC-induced diarrhea, cecal contents are less affected by this acute disturbance and better preserve the overall structure of the resident microbial community [40,41]. The E.Z.N.A. DNA Kit from Omega Bio-tek, Norcross, GA, was used to extract microbial genomic DNA from the samples in accordance with the manufacturer’s instructions. The hypervariable V3–V4 region of the bacterial 16S rRNA gene was amplified. After recovery, the PCR products were purified and then sequenced. The purified amplicons were combined in equal molar amounts and sequenced with paired-end chemistry on an Illumina MiSeq system (Illumina, San Diego, CA, USA). The sequencing services were carried out by Personalbio Technology Co., Ltd. in Shanghai, China. Microbiological data processing, functional prediction using PICRUSt, and correlation analyses were performed on the cloud computing platform offered by Shanghai Maggie Biomedical Technology Co., Ltd. (Shanghai, China). The raw sequencing data have been deposited in the NCBI SRA database under the accession number PRJNA1331971 (https://www.ncbi.nlm.nih.gov/sra/PRJNA1331971, accessed on 20 September 2025).

### 2.14. Statistical Analysis

Statistical analyses were performed using SPSS software (version 19.0). The treatment group was the independent variable; dependent variables included cell viability, NO and cytokine levels, mRNA expression of inflammatory, antioxidant, and intestinal barrier genes, body weight, mortality, diarrhea incidence, organ indices, bacterial loads, serum biochemical indices, intestinal morphology, and gut microbiota metrics. Cell viability was expressed relative to the CON group. For quantitative PCR, mRNA expression was normalized to β-actin and expressed as fold change relative to the CON group using the 2^−ΔΔCt^ method. Bacterial loads were log10-transformed before statistical analysis. For 16S rRNA sequencing data, OTU tables were rarefied to an equal sequencing depth for alpha-diversity calculation, and relative abundances (proportion of total reads per sample) were used for beta-diversity (PCoA), taxonomic composition, and LEfSe analyses. For differential microbial biomarker screening, linear discriminant analysis (LDA) was adopted within the standard LEfSe workflow [42] to quantify the effect size of each taxon and rank candidate biomarkers by group-discriminating capacity. LDA is appropriate for this purpose because it optimizes between-group separation while minimizing within-group variance, accommodating the high inter-individual heterogeneity of gut microbial relative-abundance data and providing log10 effect-size scores that prioritize biologically relevant differences beyond *p*-value-based significance alone. Comparisons among groups were performed by one-way ANOVA followed by Duncan’s multiple-comparison test. Differences were considered significant at *p* < 0.05.

## 3. Results

### 3.1. Synthesis and Characterization of AB4-OA NPs

**Figure 1:**
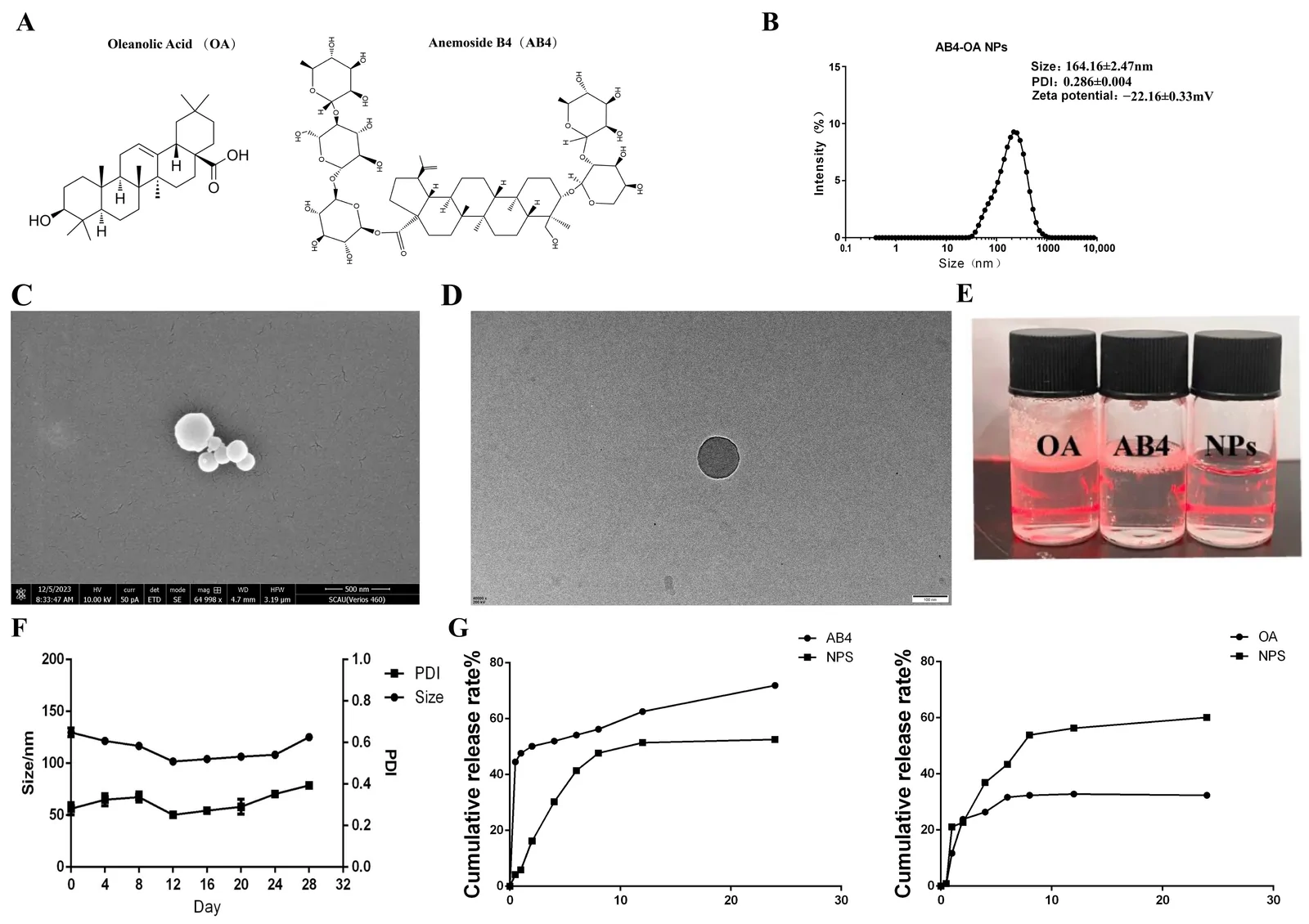
AB4 and OA formed a stable nanoassembly in aqueous solution. (**A**) Two-dimensional structures of OA and AB4; (**B**) Particle size, dispersion coefficient and potential of AB4-OA NPs; (**C**) SEM of AB4-OA NPs; (**D**) TEM of AB4-OA NPs; (**E**) Tyndall effect of AB4-OA NPs; (**F**) DLS results regarding the size and PDI of AB4-OA NPs for 28 days; (**G**) In vitro release curve of AB4-OA NPs.

According to measurements taken with a Malvern Zetasizer, the AB4-OA nanoparticles exhibited an average hydrodynamic size of 164 nm, a polydispersity index of 0.280, and a zeta potential of −22.1 mV (Figure 1B). Scanning electron microscopy (SEM) and transmission electron microscopy (TEM) imaging (Figure 1C,D) showed that the AB4-OA nanoparticles were spherical and ranged in size from 100 to 200 nm. When the supramolecular assembly in a cuvette was irradiated with a laser pointer, the dispersion system displayed a uniform and distinct light path, demonstrating a clear Tyndall effect (Figure 1E). Based on the dynamic light scattering (DLS) findings, there were no notable changes in particle size and PDI over 28 days (Figure 1F), suggesting that the AB4-OA NPs are highly stable. Standard curves for AB4 and OA were constructed by plotting concentration against peak area, both showing excellent linearity (correlation coefficient of 0.999). Based on these standard curves, the EE% were, respectively, calculated to be 70.4% and 76.7%; the DLC% were, respectively, calculated to be 67.0% and 7.3%, according to the protocol in the Section 2. In vitro release profiles at pH 7.4 demonstrated that AB4 encapsulated in AB4-OA NPs exhibited a slower release rate than free AB4 monomer, whereas OA loaded in the nanoparticles displayed a faster cumulative release compared with free oleanolic acid. These results indicate that AB4-OA NPs have a good sustained-release effect and can increase the concentration of OA in aqueous solution at the same time (Figure 1G). According to the hemolytic test results, the nanoparticles exhibited hemolytic rates of 0.53%, 1.71%, and 3.38% within the 0.5–2 mg/mL concentration range, meeting the criteria for injectable preparations (Figure S1).

### 3.2. The Self-Assembly Mechanism of AB4-OA NPs

**Figure 2:**
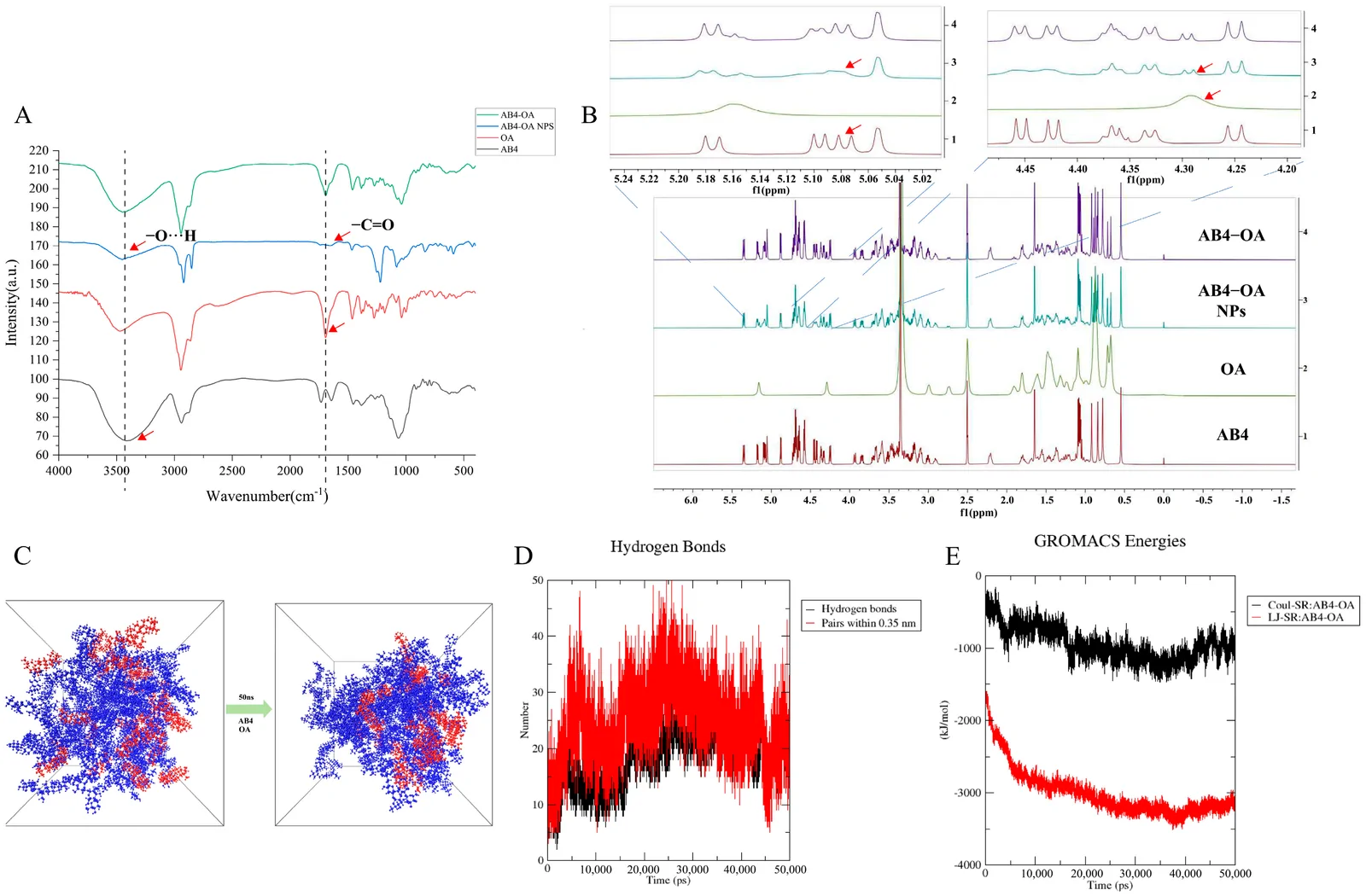
Formation mechanism of self-assembled AB4-OA NPs. (**A**) Fourier transform infrared spectroscopy (FTIR) analysis of AB4-OA NPs, with arrows indicating the hydrogen-bonded O–H stretching band (~3500 cm^−1^, –O···H) and the C=O stretching band (~1627 cm^−1^); (**B**) Nuclear magnetic resonance (NMR) analysis of AB4-OA NPs, with arrows indicating the proton signals of AB4 at 5.08–5.10 ppm and of OA at 4.28 ppm; (**C**) Structural changes of AB4-OA NPs, with AB4 is shown in blue and OA in red; (**D**) The number of hydrogen bonds; (**E**) Gromacs energies.

To understand the self-assembly process of AB4-OA NPs, Fourier transform infrared (FT-IR) spectroscopy was used to analyze molecular vibration changes, shedding light on the intermolecular interactions. In the FT-IR spectrum of the AB4 and OA physical mixture, the characteristic absorption peaks of both OA and AB4 showed no significant shift in intensity, indicating that this group was merely a simple superposition of the two. In contrast, the spectrum of the AB4-OA NPs exhibited a markedly broadened peak around 3500 cm^−1^, suggesting the presence of hydrogen bonding between the components, which reduces vibrational freedom. Concurrently, the characteristic peak of OA at 1627 cm^−1^ was significantly reduced, indicating that oleanolic acid is likely embedded within AB4, leading to attenuated C=O vibration. These findings collectively demonstrate the successful encapsulation of OA by AB4 (Figure 2A).

Analysis of Nuclear Magnetic Resonance (^1^H NMR) results (Figure 2B) revealed that the spectrum of the AB4-OA physical mixture was simply a superposition of the characteristic peaks of AB4 and OA. However, in the AB4-OA NPs group, the characteristic peak of AB4 at 5.08–5.10 ppm was markedly broadened, indicating that the hydrogen atoms on the aglycone moiety of AB4 may form hydrogen bonds with the hydroxyl groups of OA within the NPs. Furthermore, the characteristic peak of OA at 4.28 ppm changed from a singlet to a doublet, which may be attributed to different conformations of OA induced during its encapsulation by AB4. Additionally, the overall hydrogen environment of AB4 was not significantly perturbed. These results suggest that AB4 acts as a structural scaffold, interacting with specific functional groups of OA, such as hydroxyl groups, via polar groups like hydroxyl groups, to form stable aggregates.

In the simulated chamber system, aggregation among AB4, OA, and water molecules was observed after 50 ns of simulation (Figure 2C). The system exhibited an average root mean square deviation (RMSD) of 1.87 ± 0.33 nm and an average solvent accessible surface area (SASA) of 499.20 ± 42.33 nm^2^, indicating the stability of the system during the simulation (Figure S2A,B). Analysis of the radius of gyration (Rg) indicated a transition from an initially dispersed state to a compact aggregated structure, demonstrating the formation of a stable AB4-OA complex and underscoring the stability of the system throughout the simulation (Figure S2C). On average, 17.57 hydrogen bonds were maintained within the system (Figure 2D), with the number showing dynamic variation yet overall stability. Interaction energy analysis revealed that van der Waals forces (−2998 kJ/mol) constituted the dominant attractive contribution, surpassing electrostatic interactions (Coulomb energy: −939.168 kJ/mol) (Figure 2E).

### 3.3. The In Vitro Anti-Inflammatory Effect of AB4-OA NPs

**Figure 3:**
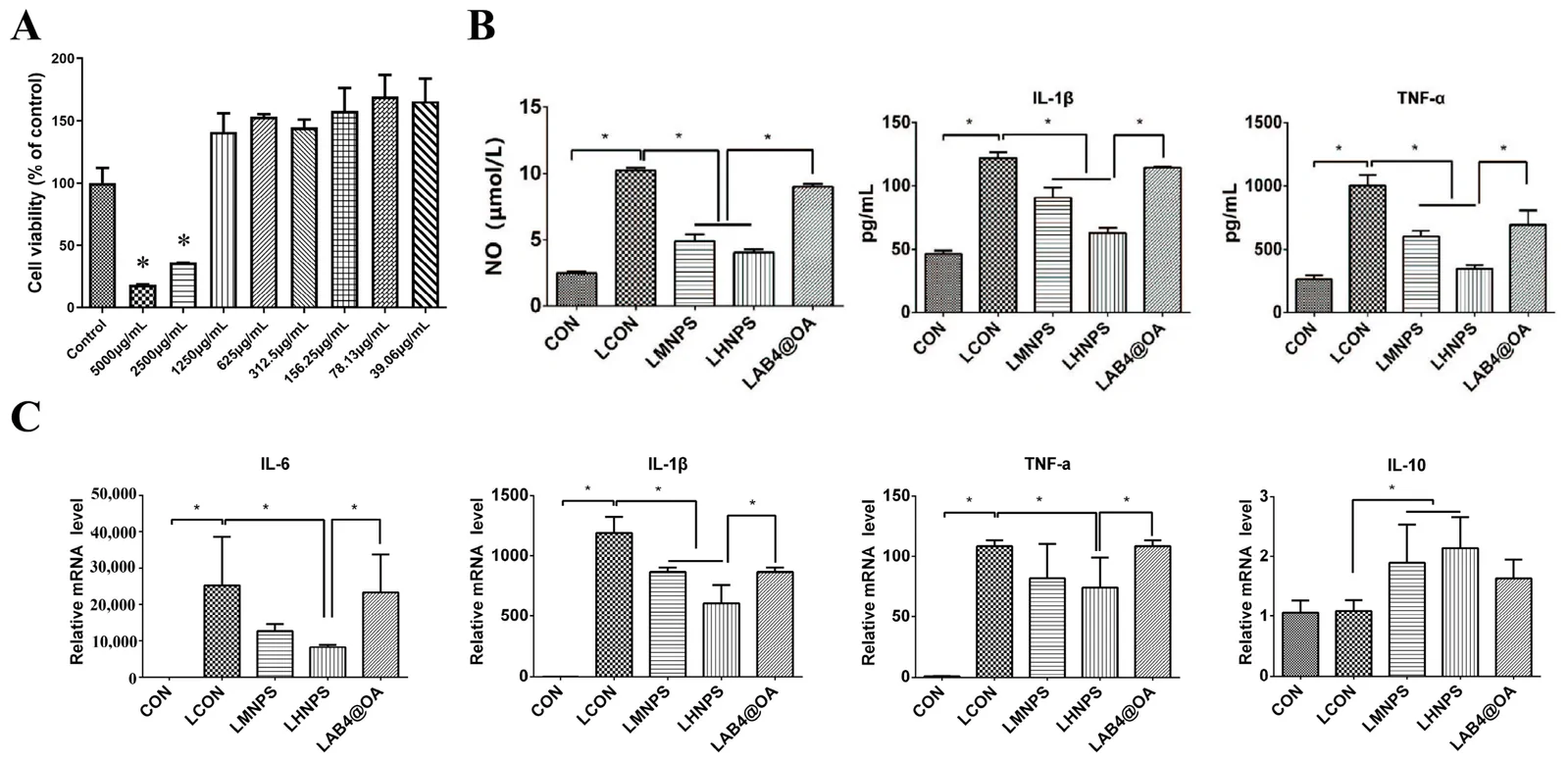
The Anti-inflammatory Activity of AB4-OA NPs in vitro. (**A**) Effects of different concentrations of AB4-OA NPs on RAW264.7 cell viability. (**B**) Effect of AB4-OA NPs on NO, IL-1β and TNF-α levels in LPS-induced RAW264.7 cells. (**C**) The mRNA expression of inflammatory genes (IL-6, IL-1β, TNF-α) and anti-inflammatory genes (IL-10) in LPS-induced RAW264.7 cells. Values are means ± sem, *n* = 3. * *p* < 0.05. Abbreviations: IL-1β, Interleukin-1β; NO, Nitric oxide; TNF-α, Tumor necrosis factor-α; IL-6, Interleukin-6; IL-10, Interleukin-10; CON, Control group; LCON, Cells stimulated with LPS only; LMNPS, LPS-stimulated cells treated with medium-dose (625 µg/mL) AB4-OA self-assembled NPs; LHNPS, LPS-stimulated cells treated with high-dose (1250 µg/mL) AB4-OA self-assembled NPs; LAB4@OA, LPS-stimulated cells treated with 1250 µg/mL physical mixture of free AB4 and OA.

The anti-inflammatory activity of AB4-OA NPs was evaluated in vitro (Figure 3). According to CCK-8 cell viability data, all tested concentrations of AB4-OA NPs ≤ 1.25 mg/mL maintained RAW264.7 cell viability above the 80% non-cytotoxic threshold, confirming no obvious cytotoxicity within this concentration range; more importantly, they also exhibited a stimulating effect on cell growth and proliferation. Compared with the control group, the LCON group exhibited significantly elevated levels of NO, IL-1β, and TNF-α in the cell supernatant (*p* < 0.05), along with markedly increased mRNA expression of IL-6, IL-1β, and TNF-α (*p* < 0.05), showing that the inflammation model was successfully established. Treatment with AB4-OA NPs significantly reversed the above parameters (*p* < 0.05) in a dose-dependent manner within the tested concentration range. Additionally, the mRNA expression of IL-10 was markedly upregulated following nanoparticle treatment. In comparison to the LAB4@OA group, AB4-OA NPs significantly decreased the levels of NO, IL-1β, and TNF-α in the cell supernatant (*p* < 0.05), as well as decreased the mRNA expression of IL-6, IL-1β, and TNF-α (*p* < 0.05).

### 3.4. The In Vitro Antioxidant Effect of AB4-OA NPs

**Figure 4:**
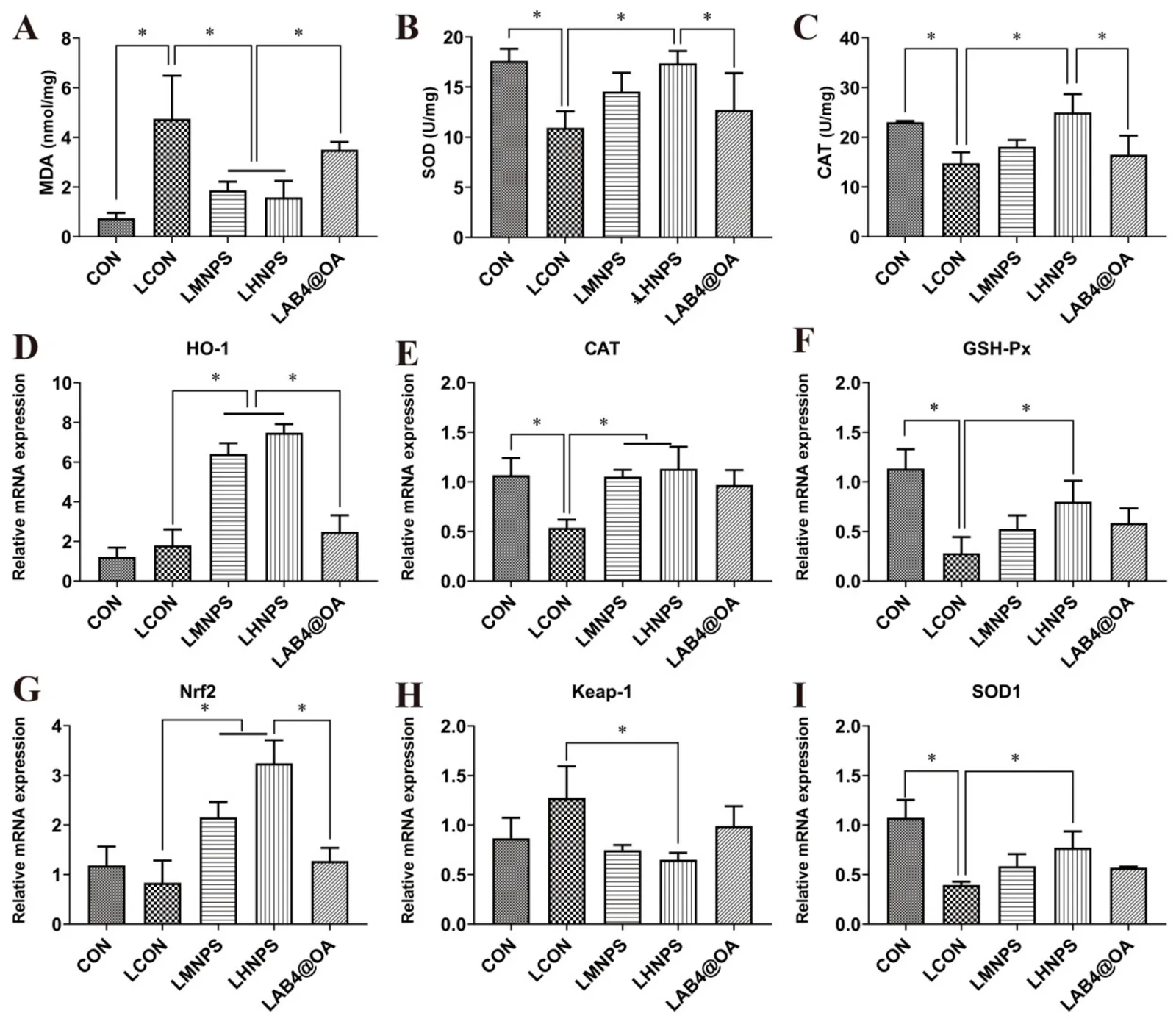
The Antioxidant Activity of AB4-OA NPs in vitro. (**A**–**C**) Effect of AB4-OA NPs on SOD, MDA, and CAT levels in LPS-induced RAW264.7 cells. (**D**–**I**) The mRNA expression of antioxidant activity genes (HO-1, Nrf2, CAT, GSH-Px, Keap-1, and SOD1). Values are means ± sem, *n* = 3. * *p* < 0.05. Abbreviations: MDA, Malondialdehyde; SOD1, Superoxide dismutase 1; CAT, Catalase; HO-1, Heme oxygenase-1; GSH-Px, Glutathione peroxidase; Nrf2, Nuclear factor erythroid 2-related factor 2; Keap-1, Kelch-like ECH-associated protein 1.

AB4-OA NPs’ antioxidant capacity was assessed in vitro (Figure 4). The LCON group had significantly higher MDA levels in the cell supernatant (*p* < 0.05) and lower SOD and CAT activity (*p* < 0.05) than the CON group. At the same time, LPS induction led to a significant rise in Keap-1 mRNA expression and a fall in antioxidant enzyme mRNA expression (SOD1, CAT, and GSH-Px). Treatment with LHNPS significantly reversed the aforementioned indicators (*p* < 0.05). Meanwhile, treatment with LMNPS and LHNPS markedly upregulated the mRNA expression of Nrf2 and HO-1 in cells, and such effects were superior to those of LAB4@OA (*p* < 0.05).

### 3.5. AB4-OA NPs Show Therapeutic Efficacy Advantages Against E. coli-Induced Enteritis

**Figure 5:**
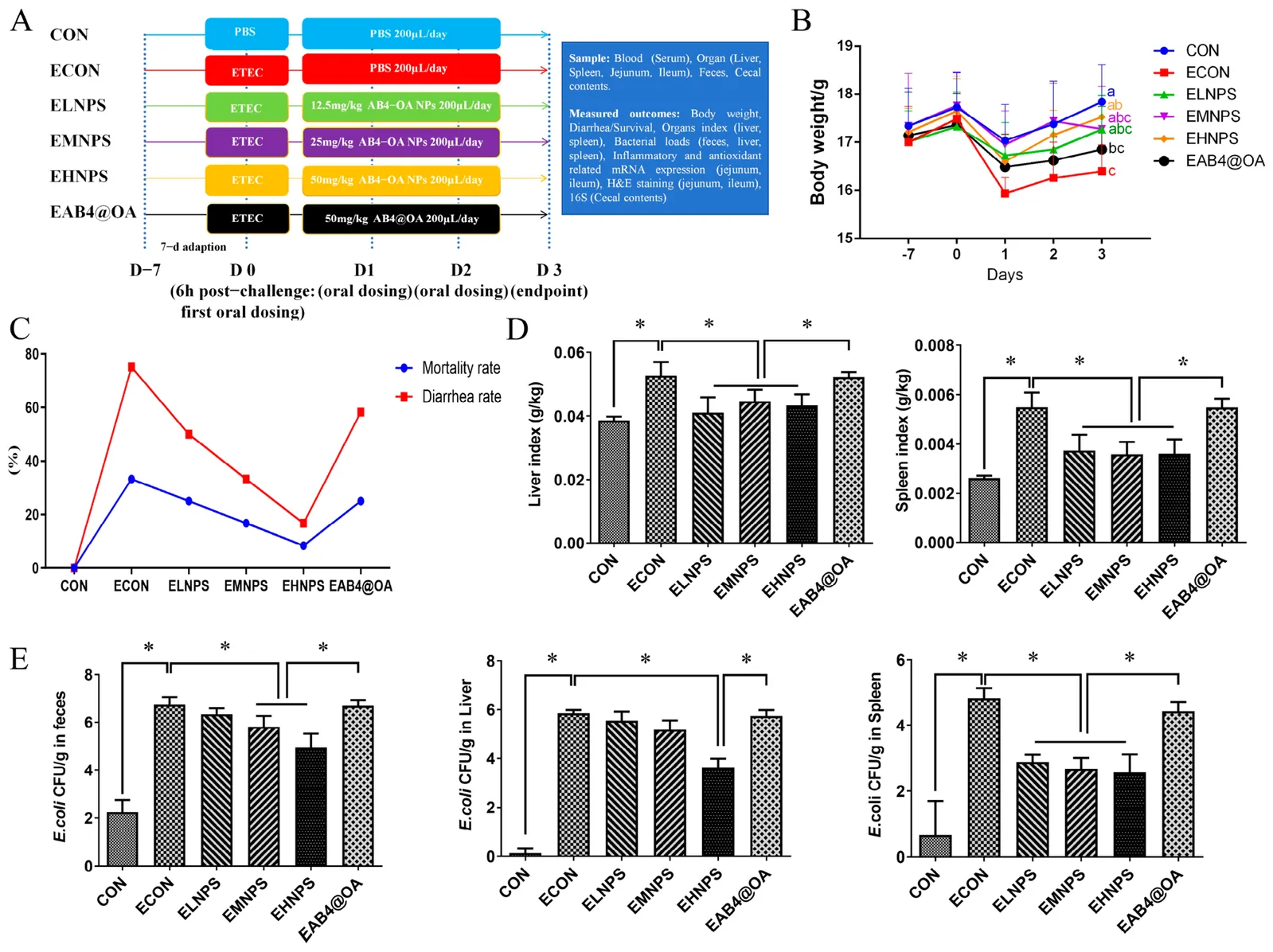
The therapeutic effect of AB4-OA NPs in vivo. (**A**) Schematic diagram of the animal experimental design. (**B**) Curve chart changes in mice’s body weight over time among the six groups. (**C**) Mortality rate and Diarrhea rate. (**D**) Liver and spleen organ index of *E. coli*-challenged mice treated with AB4-OA NPs. (**E**) Bacterial loads in fresh feces, liver and spleen. Values are means ± sem, *n* = 5. * *p* < 0.05. Abbreviations: CON, Control group; ECON, mice stimulated with ETEC only; ELNPS, ETEC-stimulated mice treated with low-dose (12.5 mg/kg) AB4-OA self-assembled NPs; EMNPS, ETEC-stimulated mice treated with medium-dose (25 mg/kg) AB4-OA self-assembled NPs; EHNPS, ETEC-stimulated mice treated with high-dose (50 mg/kg) AB4-OA self-assembled NPs; EAB4@OA, ETEC-stimulated mice treated with 50 mg/kg physical mixture of free AB4 and OA.

The effectiveness of AB4-OA NPs as a treatment was assessed in a murine model of enteritis caused by *E. coli*, with the findings displayed in Figure 5. Compared to the CON (control) group, the ECON (*E. coli*-induced model) group exhibited a significant reduction in body weight, increased mortality and diarrhea rates, a marked elevation in liver and spleen indices, and a significantly higher bacterial load of *E. coli* in the feces, liver, and spleen (*p* < 0.05). No death or obvious diarrhea was recorded in the CON group during the entire 72 h observation period. Severe diarrhea and successive animal deaths occurred in the untreated ECON group starting at 24 h post-challenge. Treatment with AB4-OA NPs resulted in a significant recovery of body weight, reduced mortality and diarrhea rates, and organ indices that were closer to those of the control group rather than the ECON group. Moreover, AB4-OA NPs greatly decreased the bacterial load in the intestines and organs, showing a more pronounced effect than the EAB4@OA group (*p* < 0.05).

### 3.6. The Effect of AB4-OA NPs on Anti-Inflammation-Related Genes in Mice Infected with E. coli

**Figure 6:**
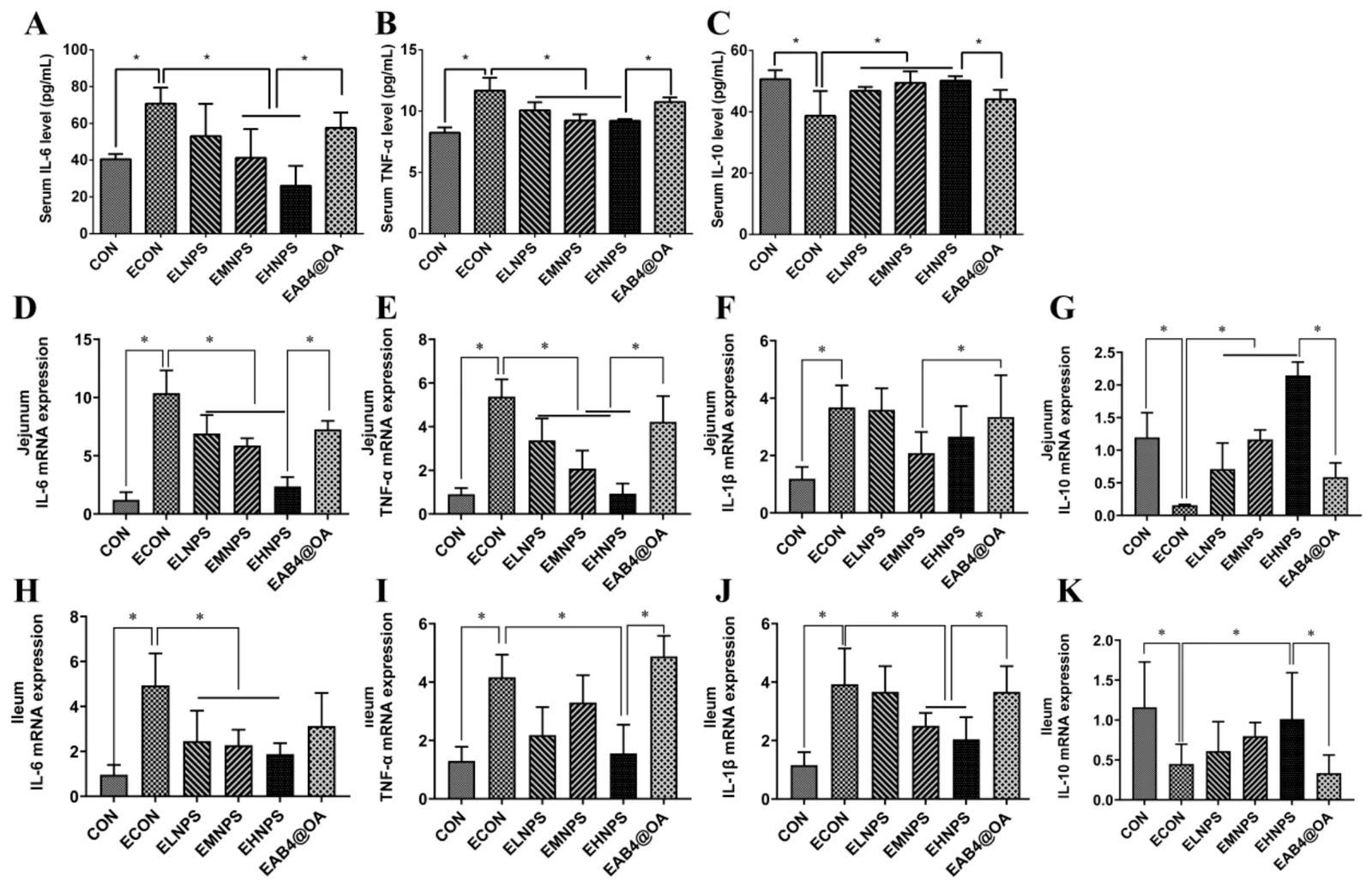
AB4-OA NPs decreased *E. coli*-induced inflammatory responses. (**A**–**C**) Serum inflammatory cytokines (IL-6, TNF-α) and anti-inflammatory cytokines (IL-10). (**D**–**K**) The mRNA expression of inflammatory-related genes (IL-6, TNF-α, IL-1β and IL-10) in the jejunum and ileum, respectively. Values are means ± sem, *n* = 5. * *p* < 0.05.

Analysis of serum inflammatory cytokines (Figure 6A) revealed that compared to the control group, the ECON group had significantly increased IL-6 and TNF-α levels, with a significant decrease in IL-10 levels (*p* < 0.05). These changes were reversed by treatment with EMNPS and EHNPS. The EHNPS group showed better results compared to the EAB4@OA group (*p* < 0.05).

Pro-inflammatory cytokine mRNA (IL-1β, IL-6, and TNF-α) was significantly higher in the jejunum of the ECON group than in the CON group, according to qRT-PCR quantitative analysis (*p* < 0.05). EHNPS treatment notably reduced the pro-inflammatory response while simultaneously boosting the mRNA expression of the anti-inflammatory cytokine IL-10 (*p* < 0.05). Treatment with EHNPS led to a significant drop in IL-6 and TNF-α mRNA levels, while IL-10 levels were elevated (*p* < 0.05). Additionally, the EHNPS group showed increased IL-10 expression along with greater downregulation of TNF-α and IL-6 in comparison to the EAB4@OA group (*p* < 0.05). Notably, the EMNPS group also showed significant suppression of IL-1β mRNA (*p* < 0.05).

In the ileum, compared to the CON group, the ECON group exhibited significantly upregulated mRNA levels of pro-inflammatory cytokines (IL-1β, IL-6, and TNF-α) and a significant downregulation of IL-10 (*p* < 0.05). The EHNPS group significantly reduced the mRNA levels of pro-inflammatory cytokines and upregulated IL-10 mRNA levels (*p* < 0.05). The EHNPS group had significantly higher levels of IL-10 mRNA (*p* < 0.05) and significantly lower levels of TNF-α and IL-1β mRNA compared to the EAB4@OA group.

### 3.7. The Effect of AB4-OA NPs on the NF-κB Pathway-Related Genes in Mice Infected with E. coli

**Figure 7:**
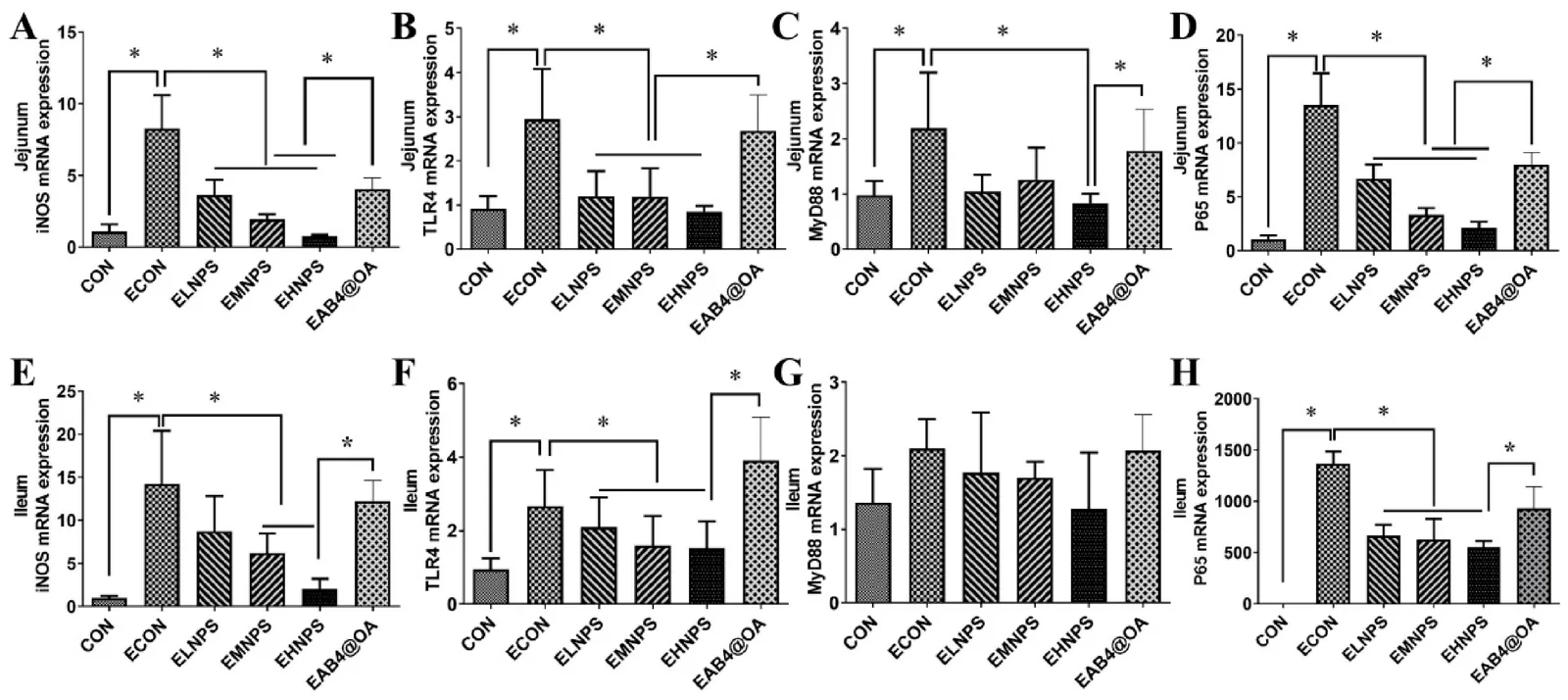
Effects of AB4-OA NPs on the inflammatory pathway. (**A**–**H**) The mRNA expression of inflammatory pathway-related genes (P65, MyD88, TLR4, and iNOS) in the jejunum and ileum, respectively. Values are means ± sem, *n* = 5. * *p* < 0.05. Abbreviations: iNOS, Inducible nitric oxide synthase; MyD88, Myeloid differentiation primary response protein 88; TLR4, Toll-like receptor 4; P65, NF-κB p65.

Our investigation subsequently evaluated the mRNA expression levels of critical signaling molecules (P65, MyD88, TLR4, and iNOS) associated with the NF-κB pathway as shown in Figure 7.

In the jejunum, there was a significant increase in the mRNA levels of P65, MyD88, TLR4, and iNOS in the ECON group compared to the CON group (*p* < 0.05). The AB4-OA NP treatment groups significantly downregulated iNOS, P65, and TLR4 mRNA levels, with the EMNPS and EHNPS groups additionally suppressing MyD88 mRNA levels (*p* < 0.05). Compared to the EAB4@OA group, the EHNPS group comprehensively inhibited the expression of iNOS, MyD88, P65, and TLR4 mRNA levels (*p* < 0.05).

In the ileum, compared to the CON group, the ECON group demonstrated a significant increase in the mRNA levels of P65, TLR4, and iNOS (*p* < 0.05). The AB4-OA NP groups significantly downregulated iNOS and P65 mRNA levels, with the EMNPS and EHNPS groups additionally suppressing TLR4 mRNA levels (*p* < 0.05). In comparison to the EAB4@OA group, the EHNPS group showed a significant decrease in iNOS, P65, and TLR4 mRNA levels (*p* < 0.05).

### 3.8. The Effect of AB4-OA NPs on the Antioxidant Pathway-Related Genes in Mice Infected with E. coli

**Figure 8:**
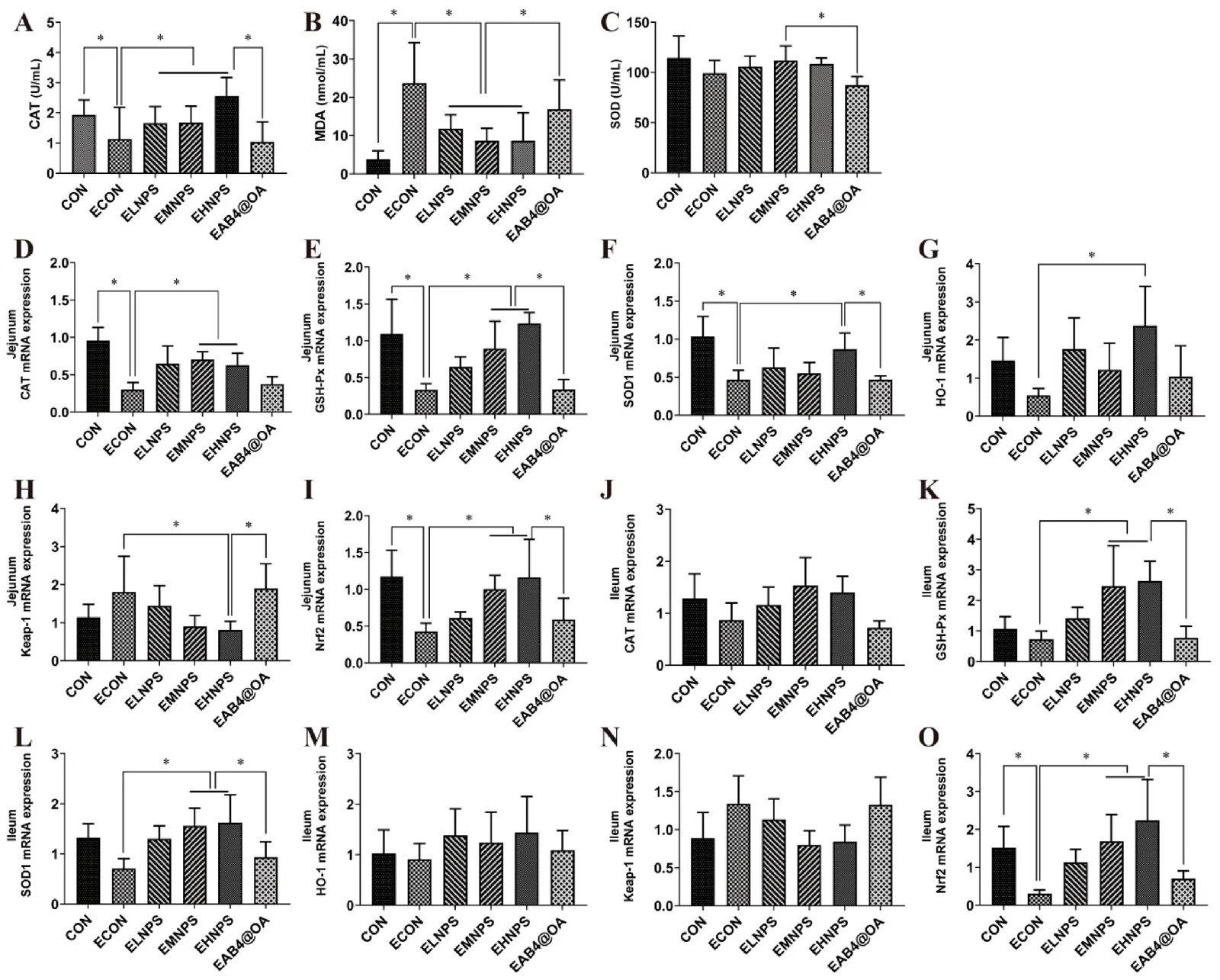
Effects of AB4-OA NPs on the antioxidant pathway. (**A**–**C**) The antioxidant levels (MDA, SOD1, and CAT) in the serum. (**D**–**O**) The mRNA expression of antioxidant pathway-related genes (Nrf2, Keap-1, HO-1, SOD1, CAT, and GSH-Px) in the jejunum and ileum, respectively. Values are means ± sem, *n* = 5. * *p* < 0.05. Abbreviations: GSH-Px, Glutathione peroxidase.

Analysis of serum antioxidant markers (Figure 8A–C) revealed that compared to the control group, the ECON group had significantly increased MDA levels, with significantly decreased activities of CAT (*p* < 0.05). These changes were reversed by treatment with EMNPS and EHNPS. The EHNPS group showed better results compared to the EAB4@OA group (*p* < 0.05).

In the jejunum, there was a significant increase in the mRNA levels of Keap-1 and a decrease in the mRNA levels of CAT, GSH-Px, SOD1, and Nrf2 in the ECON group compared to the CON group (*p* < 0.05). The EHNPS treatment group significantly reversed these aforementioned indicators (*p* < 0.05). The EHNPS group significantly increased the expression of Nrf2, SOD1, and GSH-Px mRNA levels as compared to the EAB4@OA group (*p* < 0.05).

In the ileum, compared to the CON group, the ECON group demonstrated a significant decrease in the mRNA levels of Nrf2 (*p* < 0.05). The AB4-OA NP groups significantly upregulated GSH-Px, SOD1, and Nrf2 mRNA levels (*p* < 0.05). In comparison to the EAB4@OA group, the EHNPS group showed a significant decrease in GSH-Px, SOD1, and Nrf2 mRNA levels (*p* < 0.05).

### 3.9. The Effect of AB4-OA NPs on Intestinal Permeability and Barrier-Related Genes in Mice Infected with E. coli

**Figure 9:**
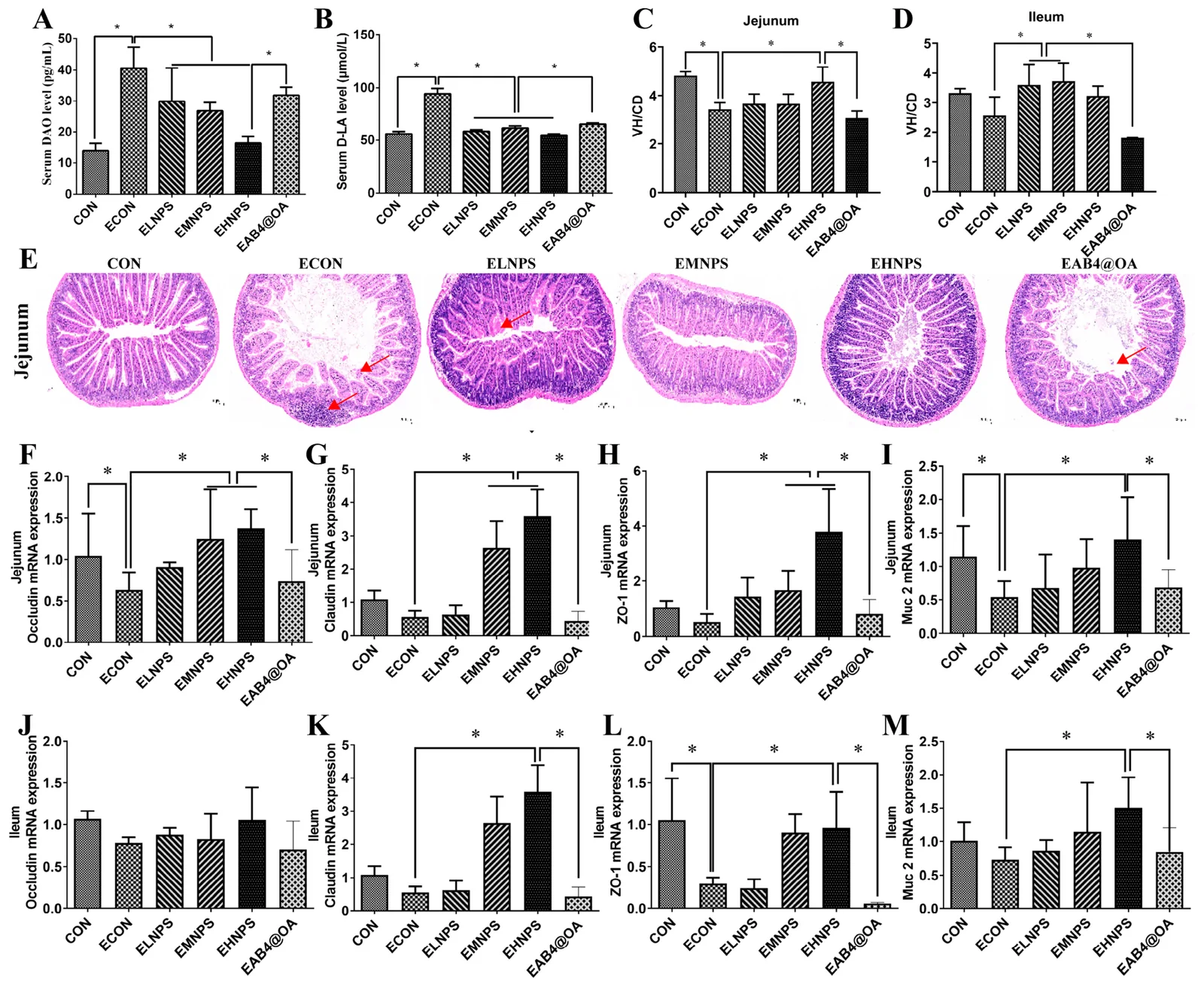
Effects of AB4-OA NPs on the intestinal mucosal barrier. (**A**,**B**) Serum intestinal permeability (DAO, D-LA). (**C**,**D**) Ratio of the villus length and crypt depth in the jejunum and ileum. (**E**) Histopathology was assessed in the jejunum, with arrows indicating inflammatory cell infiltration and intestinal epithelial debris. (**F**–**M**) The mRNA expression of intestinal mucosa barrier-related genes (Claudin-1, Occludin, ZO-1, and Muc2) in the jejunum and ileum, respectively. Values are means ± sem, *n* = 5. * *p* < 0.05. DAO, Diamine oxidase; D-LA, D-lactic acid; VH/CD, Villus height/crypt depth; Muc2, Mucin 2.

As shown in Figure 9, Serum DAO and D-LA levels were significantly elevated in *E. coli*-challenged mice, and this increase was markedly reversed by AB4-OA NP treatment (*p* < 0.05). Notably, the EHNPS group exhibited a more pronounced reduction in both markers compared with the EAB4@OA group (*p* < 0.05).

H&E staining was employed to evaluate the morphological changes in the jejunum and ileum of mice after they were challenged with *E. coli* and treated with AB4-OA NPs (Figure 9E and Figure S3). Compared to the CON group, the ECON group showed a notable decrease in villus height in all three sections of the intestine. Both EMNPS and EHNPS treatments restored villus height in these regions. The EHNPS group demonstrated superior efficacy in increasing villus height compared to the EAB4@OA group (*p* < 0.05). Furthermore, the ECON group demonstrated a reduced villus height-to-crypt depth ratio in the jejunum when compared to the CON group (*p* < 0.05). Notably, the EHNPS group significantly increased both villus height and VH/CD ratio in the jejunum (*p* < 0.05), whereas the EMNPS group elevated these parameters in the ileum (*p* < 0.05).

Using qRT-PCR, the mRNA expression of tight junction proteins (ZO-1, occludin, and claudin-1) and mucin 2 (Muc2) in the jejunum and ileum was further assessed. In the jejunum, the ECON group displayed significantly reduced mRNA expression of occludin and Muc2 compared to the CON group (*p* < 0.05). Occludin, claudin-1, and ZO-1 mRNA levels were dramatically increased by both EMNPS and EHNPS treatments (*p* < 0.05), while EHNPS additionally significantly increased Muc2 expression (*p* < 0.05). Furthermore, relative to the EAB4@OA group, EMNPS and EHNPS further increased occludin, claudin-1, and ZO-1 mRNA levels (*p* < 0.05), and EHNPS additionally promoted Muc2 expression to a significant extent (*p* < 0.05).

In the ileum, a significant downregulation of ZO-1 mRNA expression was observed in the ECON group relative to the CON group (*p* < 0.05). Significant elevation in mRNA expression of claudin-1 and ZO-1 was observed with both EMNPS and EHNPS treatments (*p* < 0.05), and the EHNPS group additionally showed increased Muc2 expression (*p* < 0.05). Moreover, in comparison to the EAB4@OA group, both the EMNPS and EHNPS groups showed a greater increase in claudin-1 and ZO-1 expression (*p* < 0.05), with the EHNPS group notably boosting Muc2 mRNA levels (*p* < 0.05).

### 3.10. Microbial Diversity and Composition of the Cecal Digesta

**Figure 10:**
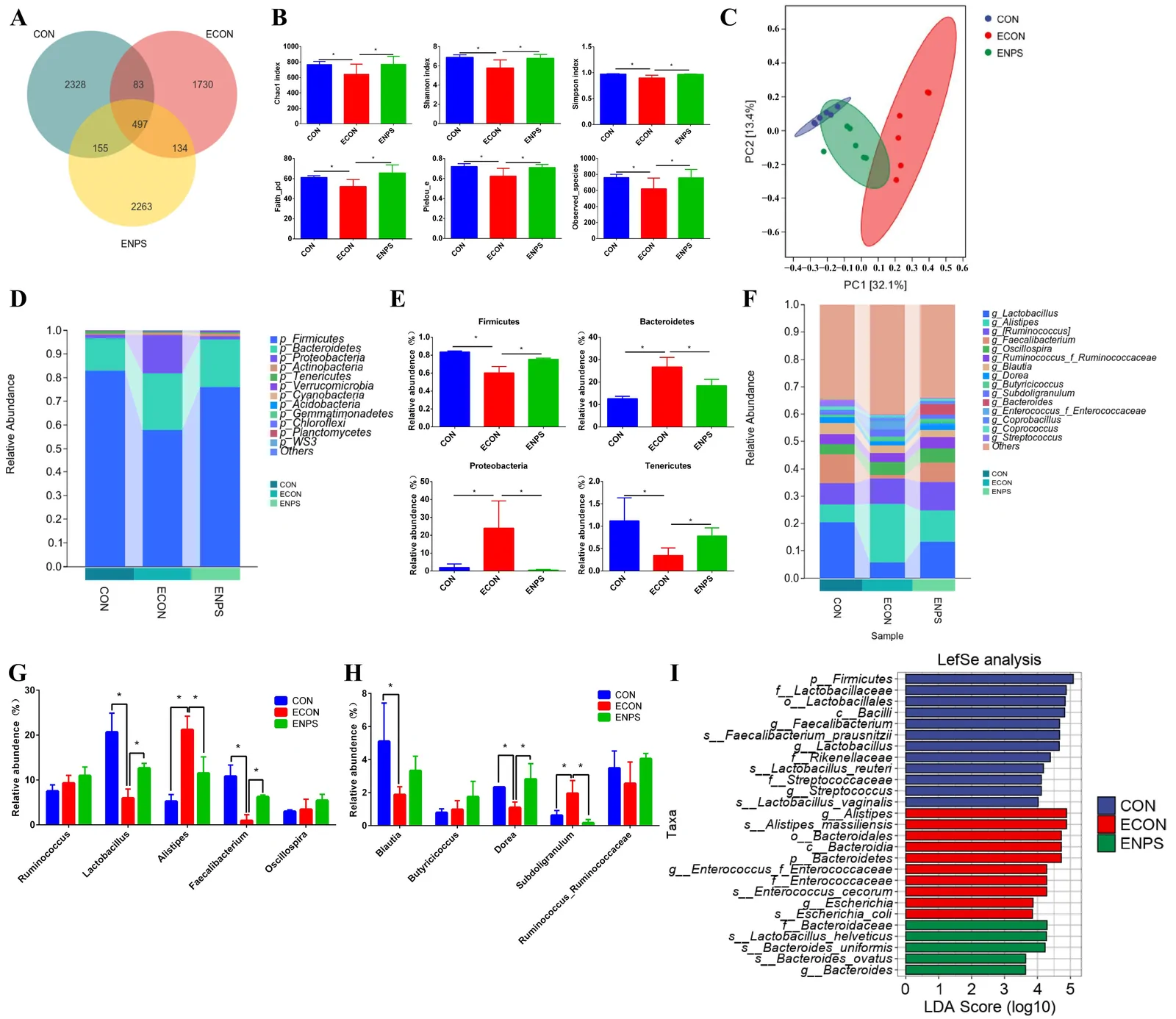
Effects of AB4-OA NPs on the microbial composition and diversity in mice infected with *E. coli*. (**A**) Venn diagram of OTU distribution across groups. (**B**) Bacterial alpha-diversity indices (Chao1, Observed_species, Shannon, and Simpson). (**C**) Principal coordinate analysis (PCoA) of microbial community structure. (**D**) Relative abundance of cecal microbiota at the phylum level. (**E**) Statistical comparison of dominant phyla. (**F**) Relative abundance of cecal microbiota at the genus level. (**G**,**H**) Statistical comparison of dominant genera. (**I**) LEfSe analysis showing differentially abundant taxa (LDA > 3). Values are means ± sem, *n* = 4. * *p* < 0.05. Abbreviations: CON, Control group; ECON, mice stimulated with ETEC only; ENPS, ETEC-stimulated mice treated with medium-dose (25 mg/mL) AB4-OA self-assembled NPs.

Venn diagram analysis identified 3063, 2444, and 3049 operational taxonomic units (OTUs) in the CON, ECON, and ENPS groups, respectively (Figure 10A). As shown in Figure 10B, the *E. coli* challenge significantly decreased the Chao 1, Shannon, Simpson, and Observed species indices (*p* < 0.05), whereas treatment with AB4-OA NPs increased these alpha diversity indices. Principal Coordinates Analysis (PCoA) demonstrated that *E. coli* introduction induced significant structural and compositional alterations in the gut microbiota (Figure 10C). The dominant bacterial taxa were primarily the phyla *Firmicutes*, *Bacteroidota*, and *Proteobacteria* at the phylum level and the genera *Lactobacillus*, *Faecalibacterium*, *Ruminococcus*, *Oscillospira*, and *Alistipes* at the genus level. Compared to the CON group, the ECON group significantly increased the relative abundances of the phyla *Bacteroidota* and *Proteobacteria* (*p* < 0.05), while significantly decreasing the relative abundances of the phylum *Firmicutes* and the genera *Lactobacillus* and *Faecalibacterium* (*p* < 0.05). The ENPS group significantly reduced the relative abundances of *Bacteroidota* and *Proteobacteria* (*p* < 0.05) and increased the relative abundances of *Firmicutes*, *Lactobacillus*, and *Faecalibacterium* (*p* < 0.05).

Linear Discriminant Analysis (LDA) Effect Size (LEfSe) was used to identify biomarkers causing intergroup differences. The CON group exhibited higher LDA scores for the phylum *Firmicutes*, the family *Lactobacillaceae*, and the genus *Faecalibacterium*. In the ECON group, the genus *Alistipes*, the family *Enterobacteriaceae*, and the genus *Escherichia* showed higher LDA scores. In the ENPS group, the family *Bacteroidaceae* and the species *Lactobacillus* helveticus demonstrated higher LDA scores.

### 3.11. Correlations Between Cecal Microbiota and Different Indicators: Spearman’s Ranks

**Figure 11:**
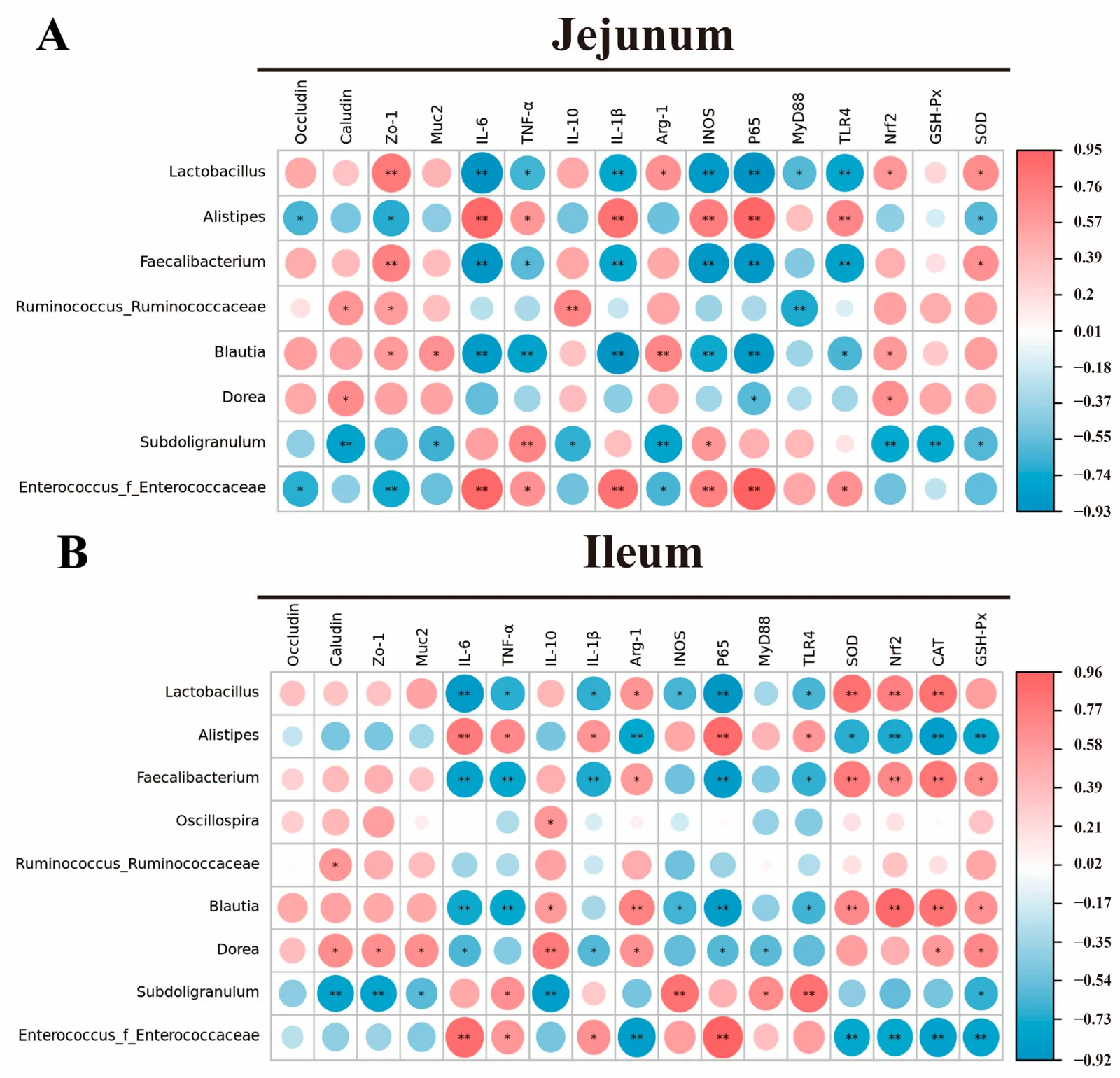
Spearman’s correlation analysis between cecal microbiota and different indicators. Values are means ± sem, *n* = 4. * *p* < 0.05, ** *p* < 0.01.

The correlation heatmap illustrates the outcomes of correlation analysis between intestinal-related genes and differential gut microbiota at the genus level in mice (Figure 11). As shown, in the jejunum, *Lactobacillus* and *Faecalibacterium* were positively associated with Nrf2 and SOD expression, and negatively correlated with the gene expression of IL-6, TNF-α, IL-1β, iNOS, P65, and TLR4. *Alistipes* was positively correlated with the gene expression of IL-6, TNF-α, IL-1β, P65, and TLR4. *Oscillospira* was positively correlated with IL-10 gene expression. *Blautia* was positively correlated with the gene expression of IL-10 and negatively correlated with the gene expression of IL-6, TNF-α, iNOS, P65, and TLR4. *Dorea* was positively correlated with the gene expression of Claudin-1, ZO-1, Muc2, and IL-10 and negatively correlated with the gene expression of IL-6, IL-1β, P65, and MyD88. *Enterococcus_Enterococcaceae* was positively correlated with the gene expression of IL-6, TNF-α, IL-1β, P65, and TLR4.

In the ileum, *Lactobacillus* and *Faecalibacterium* were positively correlated with SOD, Nrf2, CAT and ZO-1 gene expression and negatively correlated with the gene expression of IL-6, TNF-α, IL-1β, iNOS, P65, and TLR4. Alistipes was negatively correlated with the gene expression of Occludin and ZO-1 but positively correlated with the gene expression of IL-6, TNF-α, IL-1β, P65, and TLR4. *Ruminococcus_Ruminococcaceae* was positively correlated with the gene expression of Claudin-1, ZO-1, and IL-10 and negatively correlated with MyD88 gene expression. *Blautia* was positively correlated with the gene expression of IL-10 and negatively correlated with the gene expression of IL-6, TNF-α, iNOS, P65, and TLR4. *Dorea* was positively correlated with Claudin-1 gene expression and negatively correlated with P65 gene expression. *Enterococcus_Enterococcaceae* was positively correlated with the gene expression of IL-6, TNF-α, IL-1β, P65, iNOS, TLR4, and negatively correlated with the gene expression of Occludin and ZO-1.

## 4. Discussion

This study used weak-bond chemistry techniques and the compatibility theory of TCM to create Anemoside B4 (AB4) and Oleanolic Acid (OA) nanoparticles (AB4-OA NPs) using an antisolvent approach [43]. AB4 served as a natural carrier, and OA was the model drug. The physicochemical properties and performance of these NPs were systematically characterized to explore the feasibility of AB4 as a natural drug delivery vehicle. The results demonstrated that the prepared AB4-OA NPs exhibited uniform particle size, good stability, high encapsulation efficiency, and sustained release properties. After oral absorption, nanoparticles can enter the bloodstream. Determining the hemocompatibility of nanoparticles is crucial when creating them for drug delivery. The development of hemocompatible polymers significantly influences protein resistance [44]. While the negative control’s supernatant showed no red hue, the positive control’s red cells were totally ruptured when incubated with water. All of the nanoparticles’ hemolysis ratios at various doses were less than 5.0%, indicating that they would not result in hemolysis [45]. The increased hydrophilicity of the nanoparticles may lessen the contact between polymers and red blood cells, which would lessen the disruption of red blood cells [46].

FT-IR spectroscopy was employed to detect alterations in molecular vibrations and to clarify the possible self-assembly process of AB4-OA nanoparticles [47]. Post-assembly, the FT-IR spectrum of the NPs showed a markedly broadened peak around 3500 cm^−1^, suggesting potential hydrogen bonding between the components, which reduces vibrational freedom. Concurrently, the characteristic peak of OA at 1627 cm^−1^ was significantly reduced, indicating that OA is likely embedded within AB4, leading to attenuated C=O vibration. These findings collectively demonstrate the successful encapsulation of OA by AB4. Further investigations via ^1^H NMR and molecular dynamics (MD) simulations predicted the binding sites between AB4 and OA, revealing that the self-assembly formation depends on hydrogen bonding and van der Waals forces between AB4 and OA, consistent with published literature [48]. Additionally, the supramolecular complex exhibited sustained release properties and a negative surface charge; based on previous studies, these characteristics may be beneficial for targeted delivery to inflammatory sites in the intestine [49].

Macrophages, as crucial effector cells of the innate immune system, exhibit functional diversity not only in the recognition and clearance of pathogenic microorganisms but also in maintaining host antibacterial defense homeostasis through the precise regulation of the inflammatory microenvironment [50]. However, pathological stimuli can lead to macrophage overactivation, triggering uncontrolled activation of inflammatory cascades. This results in compromised intestinal mucosal barrier integrity and exacerbation of disease pathology [51]. The engagement of macrophage surface pattern recognition receptors (PRRs) by lipopolysaccharide (LPS) activates downstream signaling pathways, leading to a potent inflammatory response [52]. Consistent with our results, LPS stimulation significantly enhanced not only the release of NO, IL-1β, and TNF-α into the supernatant but also the cellular gene expression of IL-6, IL-1β, and TNF-α. The LHNPS group significantly ameliorated these alterations. These results are consistent with previous reports that AB4 and OA each suppress LPS-induced inflammatory mediator production in macrophages [53,54]. These results demonstrated that AB4-OA nanoparticles effectively attenuated the inflammatory response in LPS-stimulated macrophages. This process is particularly critical in Gram-negative bacterial infections but can cause pathological damage like sepsis if excessively activated [55]. Notably, compared with free compound formulations reported in earlier work, the AB4-OA nanoparticle system exhibited enhanced in vivo efficacy, which may be attributed to improved intestinal retention and bioavailability of encapsulated active ingredients. In contrast to the physical mixture of AB4 and OA, the co-loaded nanoparticle formulation produced greater protective effects on gut homeostasis, further supporting the advantages of the co-delivery strategy [56].

The Nrf2 pathway plays a vital role in oxidative stress and inflammation [57,58]. Under normal conditions, Nrf2 binds to Keap-1 to form a complex, in which Keap-1 facilitates the ubiquitination and degradation of Nrf2. Nrf2 separates from the complex and moves into the nucleus in response to oxidative stress, which triggers the expression of antioxidant genes. These genes include those related to oxidative stress (HO-1, SOD) [59,60]. Park et al. [61] reported that loganin alleviated LPS-induced oxidative stress in RAW264.7 cells by upregulating the protein expression of Nrf2 and HO-1. In the present study, AB4-OA NPs elevated the mRNA levels of Nrf2, as well as other downstream antioxidant enzymes, including HO-1, SOD 1, GSH-Px and CAT. Meanwhile, the mRNA expression of Keap-1 was downregulated. These findings suggest that AB4-OA NPs may suppress Keap-1 expression and upregulate Nrf2 expression, thereby increasing the production of antioxidant mediators such as HO-1, SOD 1, GSH-Px and CAT and ultimately exerting protective effects against oxidative stress. Previous studies have indicated that both Anemoside B4 (AB4) and oleanolic acid (OA) possess certain antioxidant properties [17,62]. Their conjugation into nanoparticles appears to further enhance Nrf2 activation, possibly through enhanced cellular uptake and prolonged retention. These findings are scientifically significant because they establish AB4-OA NPs as a novel, nanoparticle-enhanced platform that not only activates Nrf2-mediated antioxidant defense but also offers a practical strategy for improving the bioavailability of bioactive natural products in oxidative stress-related diseases.

Existing studies confirm that *E. coli* infection can induce intestinal inflammation, where inflamed tissues contain high concentrations of positively charged proteins (e.g., eosinophil cationic protein, transferrin). Based on previous reports [63], the negatively charged surface of AB4-OA NPs may enhance their bioadhesion to the positively charged proteins enriched in inflamed tissues through electrostatic interactions. Ex vivo experiments by Jubeh et al. confirmed this mechanism, demonstrating a two-fold greater adhesion of anionic liposomes to inflamed tissue compared to cationic or neutral liposomes [64,65]. In addition, based on the physicochemical properties of the nanoparticles and previous literature [66,67], the 160 nm particle size may confer several advantages: Firstly, it can leverage the enhanced permeability and retention (EPR) effect in intestinal epithelial cells for preferential uptake by numerous immune cells in the inflamed area; secondly, it can be transported through pores or gaps at the tips of epithelial villi via porous adsorption; thirdly, it can avoid rapid clearance caused by potentially accelerated intestinal peristalsis due to *E. coli* enteritis, thereby prolonging drug retention time at the inflammatory site, increasing drug accumulation and cellular uptake efficiency in the lesion area, and ultimately enhancing the therapeutic efficacy against *E. coli* enteritis while reducing adverse reactions. The present study demonstrates that treatment with AB4-OA NPs markedly mitigated the consequences of *E. coli* infection, including weight loss, mortality, diarrhea, and pathological impairments in the small intestine. Furthermore, their regulatory effect on inflammatory cytokine levels was superior to that of the physical mixture group, indicating enhanced in vivo efficacy of the self-assembled nanoparticles under the test conditions employed. This advantage may be attributed to the sustained-release properties and anionic surface characteristics of AB4-OA NPs, which facilitate targeted delivery to intestinal inflammatory sites, thereby enabling a more prolonged therapeutic action [68,69]. Studies by Wu et al. confirmed that AB4 significantly reduces intestinal inflammatory cytokine levels in mice with colitis [17]; investigations conducted by Chenyu Xue et al. indicated that oleanolic acid mitigates diarrhea and intestinal immune dysregulation caused by enterotoxigenic *Escherichia coli* in piglets [70]. These conclusions are consistent with our experimental results.

The intestinal barrier comprises a multilayered defense system that integrates physical, immunological, and chemical components to maintain mucosal homeostasis. Physically, the integrity of the intestinal epithelium is governed by tight junction proteins (including occludin and claudin-1), which mediate intercellular adhesion and restrict paracellular permeability, while the overlying mucus layer, primarily composed of Mucin 2 (Muc2), serves as the first line of defense against pathogen adhesion and invasion [71,72,73]. Immunologically, macrophages residing in the intestinal lamina propria play a pivotal role in tissue repair and inflammatory control, and previous studies have confirmed that *E. coli* can infect these cells and activate the NF-κB signaling pathway [74]. Research demonstrates that the EHNPS group reversed the *E. coli*-induced upregulation of iNOS, TLR4, MyD88, and NF-κB P65 at the mRNA level. Consistent with our experimental outcomes, Levistilide A inhibits the NF-κB/iNOS/NO axis, which consequently results in reduced activation of p65 and a dose-dependent decrease in iNOS expression and NO production [75,76]. Importantly, the present study further shows that AB4-OA NPs not only suppress this pro-inflammatory pathway but also restore the expression of barrier components (occludin, claudin-1, and Muc2) disrupted by *E. coli* infection, indicating a dual mode of action-inhibition of inflammation together with reinforcement of the epithelial barrier.

Compounding this inflammatory insult, oxidative stress is another prominent manifestation accompanying inflammation. An imbalance between the pro-oxidant and antioxidant systems triggers further infiltration of inflammatory cells, which in turn stimulates accumulation of lipid peroxidation, ultimately leading to oxidative damage and markedly compromising animal growth performance [77]. In this study, AB4-OA NPs significantly increased the activities of serum SOD and GSH-Px as well as the activity of CAT. Meanwhile, they upregulated the gene expression of GSH-Px and SOD1 via the Nrf2/HO-1 signaling pathway. In parallel, they upregulated the expression of occludin, claudin-1 and Muc2. Consistent with our findings, amaranth alleviates intestinal injury caused by *Escherichia coli* infection via mitigating *E. coli*-induced inflammation and improving antioxidant capacity in broilers [78]. These results indicate that AB4-OA nanoparticles can alleviate intestinal barrier damage and exert protective effects against *Escherichia coli* infection by inhibiting the activation of the intestinal NF-κB inflammatory pathway and mitigating oxidative stress.

The gut microbiota is central to host health, orchestrating key physiological processes including immune homeostasis, metabolic balance, intestinal barrier integrity, and resistance against colonization by exogenous pathogens [79]. It has been demonstrated that an *E. coli* infection causes dysbiosis of the gut microbiota [80]. In this study, the AB4-OA NPs treatment group significantly reduced the relative abundance of the phylum *Proteobacteria*; *Proteobacteria* is considered a signature phylum for gut microbiota dysbiosis, and its over-enrichment in the gut indicates host developmental abnormalities or instability in the gut microbiota structure [81]. The relative abundance of *Faecalibacterium*, *Lactobacillus*, *Blautia*, and *Dorea* was dramatically reduced in the ECON group, whereas the relative abundance of *Alistipes* and *Enterococcus_Enterococcaceae* was significantly increased. While *E. coli* infection led to a drop in beneficial bacteria and an increase in opportunistic pathogens, AB4-OA NPs considerably improved the gut microbiota imbalance brought on by *E. coli* infection. These changes recapitulate the established signature of *E. coli*-induced dysbiosis, particularly the expansion of Proteobacteria and the depletion of beneficial taxa such as *Faecalibacterium* [82]. Treatment with AB4-OA NPs partially reversed this dysbiosis by restoring *Faecalibacterium*, *Lactobacillus*, *Blautia*, and *Dorea* and reducing harmful bacteria, in line with recent reports that nanosystems can rebalance the gut microbiota in enteritis models [83,84]. Because the restoration of beneficial taxa was associated with improved barrier and inflammatory markers, microbiota modulation likely contributes to–rather than merely accompanies—the therapeutic efficacy of AB4-OA NPs.

The current research has primarily centered on the preparation of the nanoparticles and their pharmacodynamic evaluation. Future investigations on the nanoparticle properties will require comprehensive pharmacokinetic studies, in vivo acute and chronic toxicity tests, as well as distribution studies, to thoroughly evaluate the alterations in properties before and after nano-formulation.

## 5. Study Limitations and Future Directions

This study fully elucidates the underlying mechanism by which AB4-OA nanoparticles treat *Escherichia coli*-induced enteritis in mice, yet several limitations still exist. First, this study confirmed that the Nrf2 and NF-κB pathways are coordinately regulated by the nanoparticles, which only demonstrates a correlative relationship between the two pathways. Further validation of the causal link between these pathways and therapeutic efficacy via pathway inhibitors, gene knockout/overexpression, and other approaches remains an essential direction for subsequent research.

Second, although both physicochemical characterization and pharmacodynamic data revealed that the nanoparticles exert superior effects relative to the physical mixture of the free drugs, this work lacks pharmacokinetic, tissue distribution, and ADME analyses. Therefore, we cannot provide direct evidence for improved bioavailability and intestinal targeting efficiency of the nanoformulation. Follow-up studies should adopt plasma drug concentration monitoring, tracer imaging, and other techniques to quantitatively evaluate the in vivo behavior of this delivery system.

Third, AB4-only and OA-only treatment groups were not included in the present study. Therefore, the greater therapeutic effects of AB4-OA NPs relative to the physical mixture cannot be interpreted as evidence of pharmacologic synergism between AB4 and OA.

Fourth, an AB4-OA NPs-alone group (i.e., NPs administration without LPS/ETEC stimulation) was not included in the present experimental design, which limits our ability to definitively exclude the possibility that the nanoparticles themselves directly interfere with macrophage function through TLR4/MyD88 signaling. Although our preliminary experiments indicated that AB4-OA NPs alone did not significantly affect basal macrophage viability, the inclusion of such a control group remains necessary to rigorously rule out non-specific immunomodulatory effects. We plan to address this limitation in our future studies.

Fifth, only male mice were enrolled for short-term experiments in the present study. Supplementary assessments are required regarding the long-term toxicity, biocompatibility and immunogenicity of the nanoparticles, as well as gender-related differences in their pharmacodynamic and toxicological profiles.

In addition, first-line clinical drugs including dexamethasone and enrofloxacin were not included as control groups in the experiment, making it difficult to objectively evaluate the clinical therapeutic advantages of this formulation. Future research is recommended to establish positive drug control groups for horizontal efficacy comparison, followed by sequential large-animal experiments and standardized trials, so as to ultimately determine the safety threshold and optimal dosing regimen of the nanoformulation for application in poultry and livestock.

## 6. Conclusions

In this study, the natural compounds AB4 and OA were found to self-assemble into carrier-free nanoparticles (AB4-OA NPs). Spectral analyses, including FTIR, NMR, and molecular dynamics simulations, confirmed the self-assembly behavior and elucidated the underlying interaction mechanisms. To confirm the nanoformulation’s in vivo performance, we further assessed the therapeutic effectiveness of AB4-OA NPs and each of their constituent parts in a mouse model of *E. coli*-induced enteritis. AB4-OA NPs showed better results in regulating inflammatory cytokines, modulating antioxidant enzyme activities, reestablishing tight junction protein expression, and rebalancing gut microbiota than the physical mixture. These advantages may be attributed to their nanoscale size and anionic surface properties, which may enhance bioavailability and targeting capability based on previous studies; however, these parameters were not directly measured in the present study and warrant further investigation. This study not only proposes a novel therapeutic strategy for bacterial enteritis but also deciphers the mechanism of this herbal pair, providing new perspectives for understanding TCM active constituents and offering mechanistic insights into the scientific basis of TCM compatibility.

## Data Availability

The original contributions presented in this study are included in the article/Supplementary Material. Further inquiries can be directed to the corresponding author.

## Supplementary Materials

The following supporting information can be downloaded at: https://www.mdpi.com/article/10.3390/antiox15091081/s1, Figure S1: Hemolytic test analysis. Values are means ± sem, n = 3. Abbreviations: DDW, Double-distilled water; Figure S2: Molecular dynamics simulation of AB4-OA NPs. (A) Root Mean Square Deviation (RMSD); (B) Solvent accessible surface area (SASA); (C) Time evolution of the radius of gyration for the AB4-OA NPs system during the simulation time; Figure S3: Effect of AB4-OA NPs on jejunm and ileal morphology in mice infected with *E. coli* by H&E staining (40×). (A) Histopathology were determined in ileum; (B) Ratio of the villus length in jejunum and ileum; (C) Ratio of the crypt depth in jejunum and ileum. Table S1: The gene sequence for RT-qPCR.
