# Supplementary material for: Self-Assembled Carrier-Free Nanostructures of Anemoside B4 and Oleanolic Acid Alleviate Intestinal Injury Induced by Intraperitoneal Escherichia coli Challenge in Mice
Source: Antioxidants (Basel). 2026 Aug 28;15(9):1081. doi: 10.3390/antiox15091081 (PMC13603956; doi:10.3390/antiox15091081)
Supplement: Supplementary file 1 [file antioxidants-15-01081-s001.zip › antioxidants-4415362-supplementary.pdf]

SUPPLEMENTARY MATERIAL

Table S1. The gene sequence for RT-qPCR

Gene	Direction	Sequence (5′ - 3′)	Accession number
β-actin	Forward	GCTCTTCCAGCCCTCCTTCC	NM_007393.5
	Reverse	ACAGCACCGTGTTGGCGTAG	
IL-10	Forward	CTATGCTGCCTGCTCTTACTGACTG	XM_036162094.1
	Reverse	GAGTCGGTTAGCAGTATGTTGTCCAG	
IL-1β	Forward	AGGCAGGCAGTATCACTCATTG	XM_006498795.5
	Reverse	CGTCACACACCAGCAGGTTATC	
IL-6	Forward	TCCAGTTGGCTTCTTGGGAC	NM_031168.2
	Reverse	TCCTTTCCGGACTTGTGGAG	
TNF-α	Forward	CTGGGACAGTGACCTGGACT	NM_013693.3
	Reverse	GCACCTCAGGGAAGAGTCTG	
TLR4	Forward	GCCATCGCTGCTAACATCATC	NM_021297.3
	Reverse	CTCATACTCAAAGATACACCATCGG	
MyD88	Forward	CTAGAGCTGCTGGCCTTGTT	NM_010851.3
	Reverse	TCATCAAGGGTGGTGATGCC	
INOS	Forward	CTGCCAGGGTCACAACTTTAC	NM_010927.4
	Reverse	CAGCTCAGTCCCTTCACCAA	
Occludin	Forward	ACGGACCCTGACCACTATGA	NM_001360539.1
	Reverse	TCAGCAGCAGCCATGTACTC	
ZO-1	Forward	GATGAGCGGGCTACCTTA	XM_006540779.5
	Reverse	TGGAGACTGCGTGGAATG	
Claudin-1	Forward	TGGCCACGTCATGGTATGG	NM_016674.4
	Reverse	AACGGGTGTGAAAGGGTCATAG	
Mucin-2	Forward	GCTGCACCAAGAACATCTCCA	NM_023566.4
	Reverse	TTCCAGGGTAACACTTCGGACAC	
P65	Forward	AGGCTTCTGGGCCTTATGTG	NM_009045.5
	Reverse	TGCTTCTCTCGCCAGGAATAC	
SOD1	Forward	GAGCGGGCCAGTAAAGGTTA	NM_205064.155
	Reverse	CCCTTTGCAGTCACATTGCC	
CAT	Forward	GCCACATGGTGACTACCCTC	NM_001031215.2
	Reverse	TGTTGCTAGGGTCATACGCC	
GSH-Px	Forward	GACCAACCCGCAGTACATCA	NM_001277853.2
	Reverse	GAGGTGCGGGCTTTCCTTTA	
Keap-1	Forward	AGCAGCGTGGAGAGGTATGA	KU321503.1
	Reverse	GCGTACAGCAGTATGTTCAGC	
HO-1	Forward	ACGAGTTCAAGCTGGTCACG	NM_205344.1
	Reverse	GGATGCTTCTTGCCAACGAC	
Nrf2	Forward	CGCTTTCTTCAGGGGTAGCA	NM_205117.1
	Reverse	AGTTCGGTGCAGAAGAGGTG	

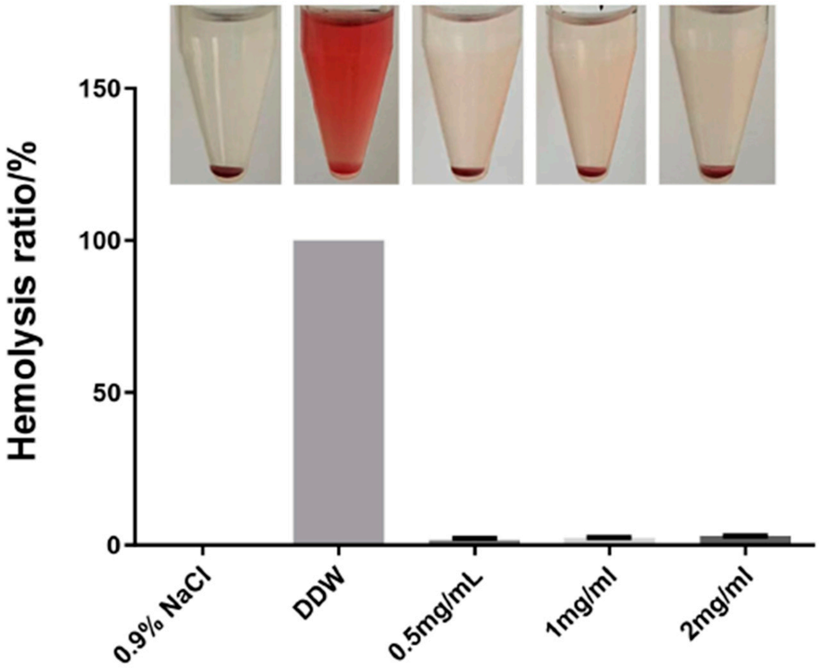

Figure. S1. Hemolytic test analysis. Values are means ± sem, n = 3. Abbreviations: DDW, Double-distilled water.

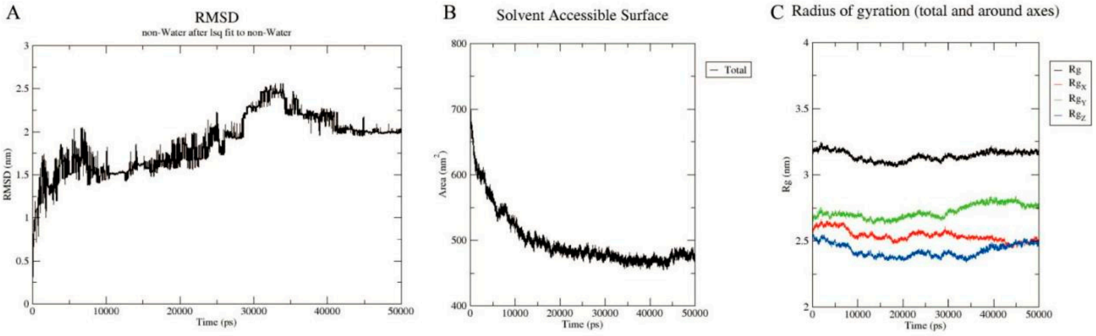

Figure. S2. Molecular dynamics simulation of AB4-OA NPs. (A) Root Mean Square Deviation (RMSD); (B) Solvent accessible surface area (SASA); (C) Time evolution of the radius of gyration for the AB4-OA NPs system during the simulation time.

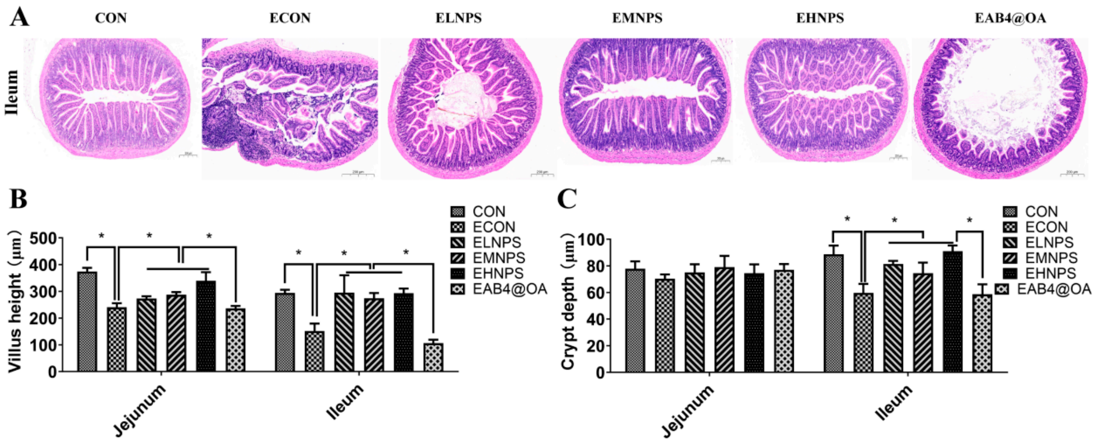

Figure. S3. Effect of AB4-OA NPs on jejunm and ileal morphology in mice infected with *E. coli* by H&E staining (40×). (A) Histopathology were determined in ileum; (B) Ratio of the villus length in jejunum and ileum; (C) Ratio of the crypt depth in jejunum and ileum.
